# Synthesis of complex unnatural fluorine-containing amino acids

**DOI:** 10.1016/j.jfluchem.2020.109630

**Published:** 2020-11

**Authors:** William D.G. Brittain, Carissa M. Lloyd, Steven L. Cobb

**Affiliations:** Department of Chemistry, Durham University, Durham DH1 3LE, United Kingdom

**Keywords:** Amino acids, Fluorination, Synthetic methods, Peptide chemistry

## Abstract

The area of fluorinated amino acid synthesis has seen rapid growth over the past decade. As reports of singly fluorinated natural amino acid derivatives have grown, researchers have turned their attention to develop methodology to access complex proteinogenic examples. A variety of reaction conditions have been employed in this area, exploiting new advances in the wider synthetic community such as photocatalysis and palladium cross-coupling. In addition, novel fluorinated functional groups have also been incorporated into amino acids, with SF_X_ and perfluoro moieties now appearing with more frequency in the literature. This review focuses on synthetic methodology for accessing complex non-proteinogenic amino acids, along with amino acids containing multiple fluorine atoms such as CF_3_, SF_5_ and perfluoroaromatic groups.

## Graphical abstract

**Figure:**
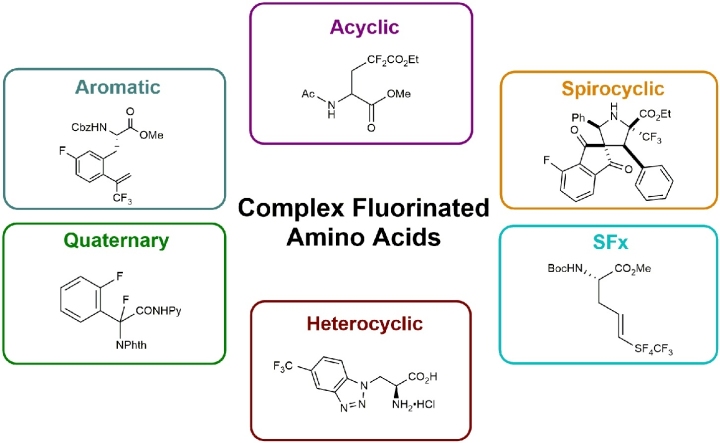

## 1 Introduction

The introduction of fluorine atoms into molecules to modulate their physical and chemical properties has become a mainstay in many research areas in modern chemistry [[1], [2], [3], [4], [5], [6], [7]]. This strategy has proven to be remarkably effective in terms of drug discovery, and in 2010 > 20 % of all pharmaceuticals on the market contained one or more fluorine atoms with this trending upwards to around 30 % today [4]. Fluorine and hydrogen atoms are similar in size, but the former possess a much greater electronegativity. This means that introduction of a fluorine will not greatly increase the steric bulk of a given molecule, but it can have profound electronic implications. This can in turn lead to significant changes in a molecule’s chemical and physical properties. One added benefit to the introduction of fluorine atoms is that they are spin-active and thus provide a handle for spectroscopic analysis through ^19^F NMR. Fluorine NMR is a remarkably sensitive technique and displays characteristic chemical shifts and coupling constants [8]. This can be especially useful in biological systems in which fluorine atoms are not naturally occurring and ^1^H NMR analysis can be complex.

The introduction of fluorine atoms into amino acids can be particularly advantageous. The introduction of fluorine atoms into peptides and proteins has been shown to increase their enzymatic stability and can also modulate their activities. Fluorinated amino acids have also seen widespread use as biological probes due to their unique spectroscopic properties. With many reported syntheses of natural and unnatural fluorine-containing amino acids currently in the literature, this review specifically focuses on the construction of complex fluorine-containing amino acids, particularly those published after 2010. It will provide examples of non-proteinogenic fluorinated amino acids, with an emphasis on non α-amino acids, and amino acids containing multiple fluorine atoms such as CF_3_, SF_5_ or perfluoroaromatics. The selection of material presented has also been carried out so that this review is complementary to the excellent general reviews in the area including those by Sutherland et al. [9], Qiu et al. [10], Hunter et al. [11] and more recently, Koksch and co-workers [12].

## 2 Complex fluorine-containing aromatic amino acids

Unnatural amino acids containing aromatic rings have been the subject of many synthetic studies due to the range of functionality and substitution patterns that can be targeted. Aromatic residues introduced into peptides and proteins have been used for a variety of applications from quantitative analysis [13,14] to fluorescence [15,16] and tagging [17]. Fluorinated aromatic amino acids are valuable tools for probing biosynthetic pathways, as they can be easily monitored by ^19^F NMR [18]. In addition to their spectroscopic properties, fluorinated aromatic amino acids have been shown to greatly change the properties of peptides, including their propensity to form helices [19], protein-protein interactions [20] and their solubility profiles [21]. Due to their wide-ranging applications, expanding the library of fluorinated aromatic amino acids has seen increasing attention from the synthetic community.

### 2.1 Aromatic amino acids

**Scheme 1:**
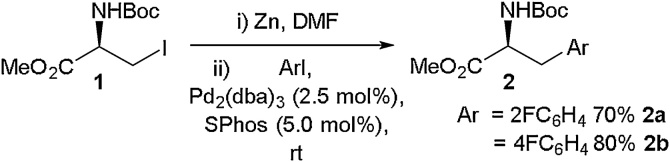
Negishi cross-coupling in the synthesis of α-amino acids, Jackson and co-workers [30].

**Scheme 2:**
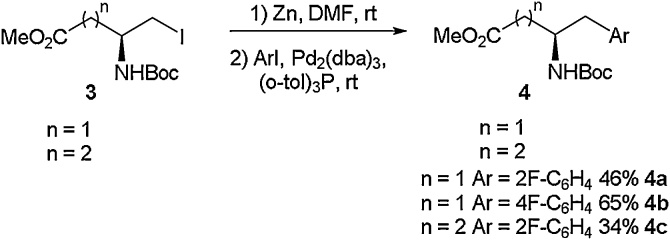
Negishi cross-coupling in the synthesis of β- and γ-amino acids, Jackson and co-workers [23].

The development of cross-coupling strategies to take advantage of halo-amino acid precursors has allowed access to a wide variety of fluorinated unnatural amino acids. Negishi cross-coupling of halo-serine derivatives is a mainstay in this area and was pioneered by the Jackson group [[22], [23], [24], [25], [26], [27], [28], [29], [30], [31]]. The Negishi cross-coupling reaction allows two, often readily available, halogen containing building blocks to be coupled together through a halo-zinc intermediate in the presence of palladium. Using this reaction and an iodo-serine derivative (**1**), Jackson was able to prepare a range of aromatic α-amino acids, and in particular, several fluorinated examples in excellent yields (Scheme 1) [30]. Jackson also employed the same approach to access fluorinated β- and γ-amino acids in moderate to good yields (Scheme 2) [23].

**Scheme 3:**
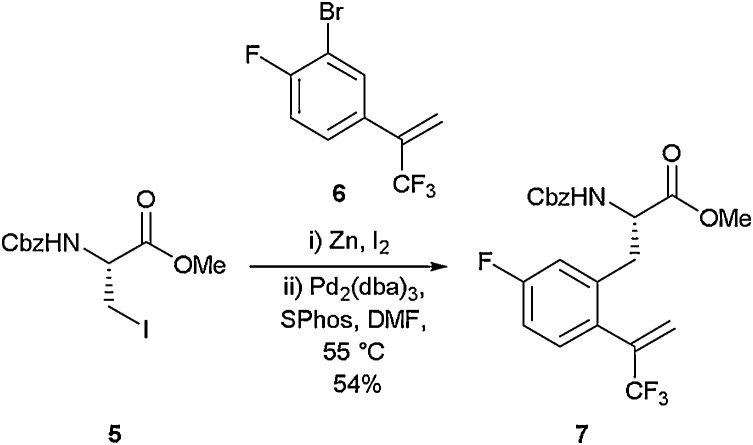
A fluorinated amino acid used in the synthesis of an oxidation catalyst, Featherston and Miller [34].

As the coupling partner in this reaction is a simple halogenated aryl species, it allows this approach to be widely implemented in a modular fashion to rapidly build amino acid libraries. This highly modular approach has been exploited by other researchers to access a range of fluorinated motifs [32,33]. For example, Featherston and Miller employed a Negishi coupling strategy to access the multiple-fluorine containing amino acid **7** [34]. Starting from an iodo-serine derivative (**5**) and a bromoaromatic coupling partner (**6**), Negishi cross-coupling was carried out to access amino acid **7** in 54 % yield (Scheme 3). This amino acid was employed as a critical building block in the synthesis of a peptide-based oxidation catalyst.

Negishi coupling is not the only cross-coupling reaction which has been employed to access aromatic fluorinated amino acids.

**Scheme 4:**
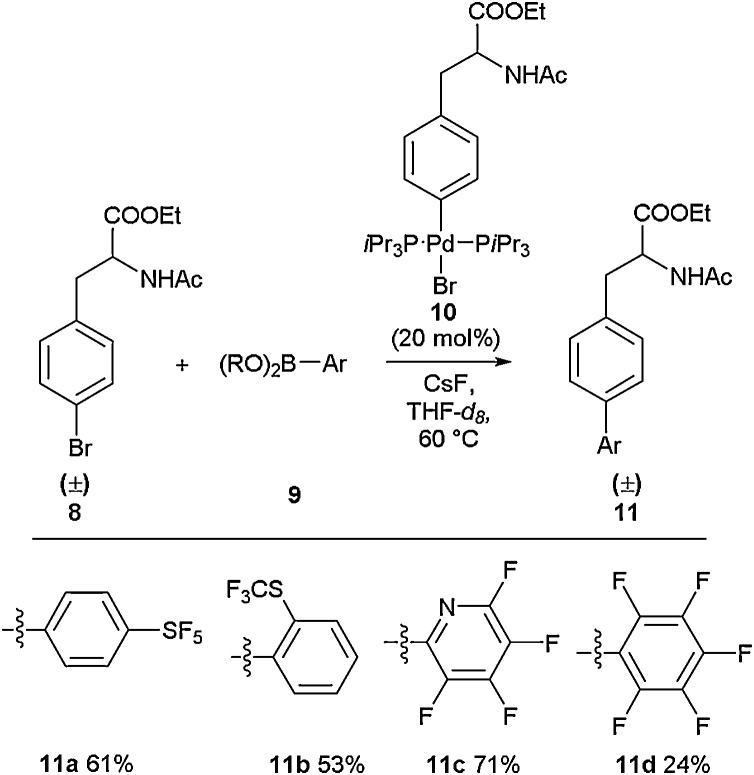
Suzuki-Miyaura cross-coupling in the synthesis of highly fluorinated amino acids, Herrmann and co-workers [35].

A range of multiple fluorine-containing bi-aryl amino acids including perfluoroaromatic examples were synthesised *via* a Suzuki approach by Herrmann and co-workers as part of a larger mechanistic study [35]. Bi-aryl SF_5_ **11a** and SCF_3_ **11b** amino acids were accessed in 61 % and 53 % yields, respectively, alongside tetrafluoropyridyl **11c** and pentafluorophenyl **11d** examples (Scheme 4). From their NMR-based mechanistic studies it was concluded that the oxidative addition step of the cross-coupling was not rate-limiting and that the reaction proceeded *via* boronate intermediates.

**Scheme 5:**
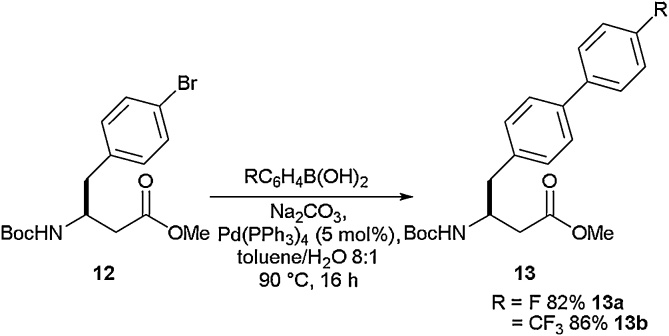
Suzuki-Miyaura cross-coupling to access bi-aryl β-amino acids, Seebach and co-workers [36].

Singly fluorinated and trifluoromethyl-appended bi-aryl β-amino acids were reported by Seebach and co-workers using Suzuki cross-coupling as a key synthetic step (Scheme 5) [36]. In this case, a free boronic acid was coupled to a bromo-β-phenylalanine derivative to give bi-aryl β-amino acids. Here, fluorinated bi-aryl amino acid **13a** was synthesised in 82 % yield and the 4-CF_3_ bi-aryl β-amino acid **13b** was obtained in 86 % yield.

**Scheme 6:**
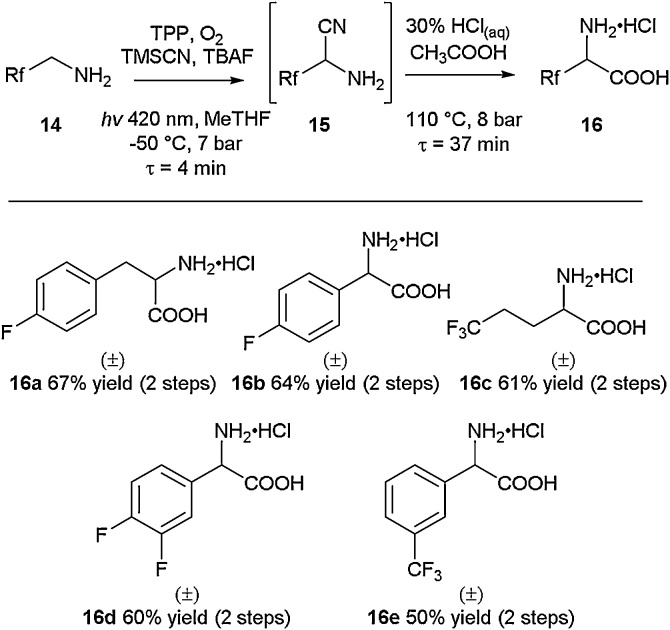
Flow synthesis of fluorinated amino acids, Seeberger and co-workers [37].

The implementation of flow techniques has helped revolutionise the way some synthetic routes are tackled, allowing for precise reaction mixing that cannot be achieved using batch approaches. Seeberger and co-workers described the rapid synthesis of fluorinated α‐amino acids through the utilisation of flow technology in 2015 (Scheme 6) [37]. Amine **14**, possessing varying fluorinated aliphatic, benzylic and homobenzylic functionalities, was converted to the intermediate α‐amino nitrile species **15** through photooxidative cyanation according to conditions similar to those already described in the literature [38]. Further flow hydrolysis under acidic conditions led to the isolation of the desired fluorinated α‐amino acids **16a-16e** without the need for chromatographic purification in yields ranging from 50 to 67 % (over two steps).

**Scheme 7:**
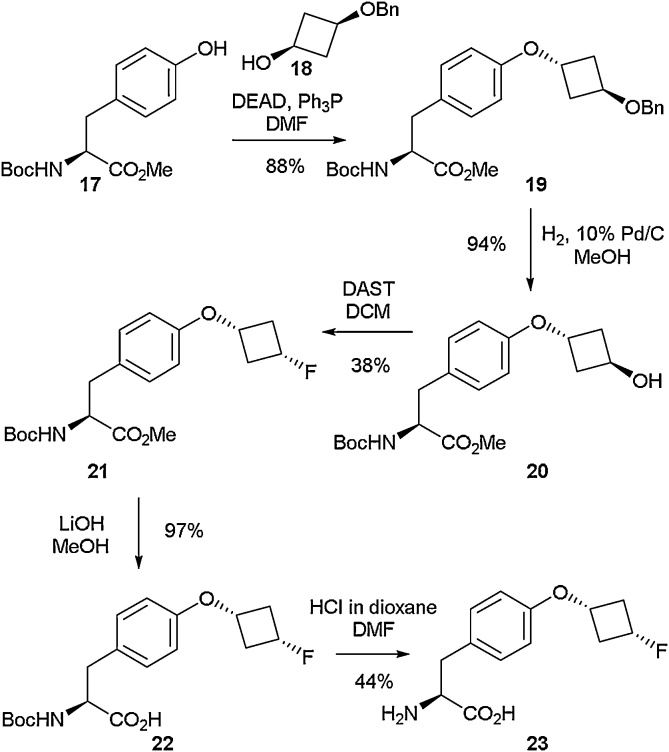
Synthesis of *O*-(*cis*-3-fluorocyclobutyl)-l-tyrosine, Graham and co-workers [39].

The synthesis of *O*-(*cis*-3-fluorocyclobutyl)-l-tyrosine (**23**) was detailed in 2013 by Graham and co-workers (Scheme 7) [39]. The first step in the process employed a Mitsunobu reaction [40] between cyclobutanol derivative **18** and Boc-L-Tyr-OMe **17** with a yield of 88 %. Further exposure to hydrogenation conditions in order to remove the benzyl protecting group followed by fluorination of the hydroxyl with DAST, resulted in successful isolation of **21**. The methyl ester and Boc protecting groups could then be removed [41] sequentially in order to generate amino acid **23**. The same methodology was successfully applied for accessing the analogous d-isomer of **23**. A fluorine-18 radiolabelled analogue of **23** was also isolated in a low-yielding two-step process and was shown to be a promising PET imaging agent for the evaluation of biological activity.

### 2.2 Quaternary aromatic fluorinated amino acids

**Scheme 8:**
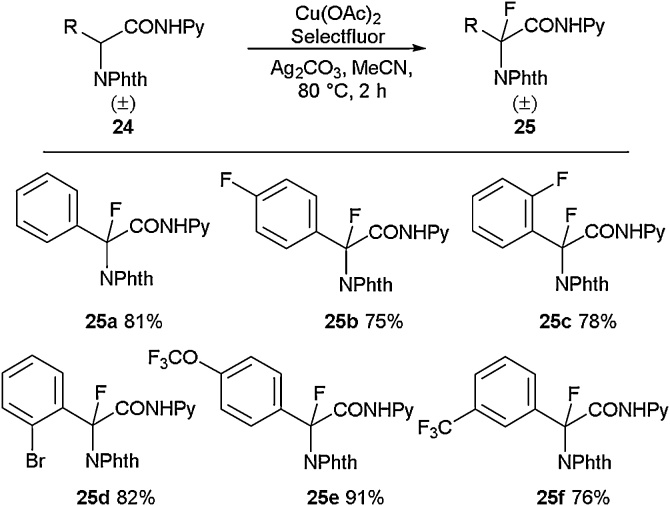
Copper-catalysed fluorination of C—H bonds to access quaternary fluorinated amino acids, Liu and co-workers [42].

**Scheme 9:**
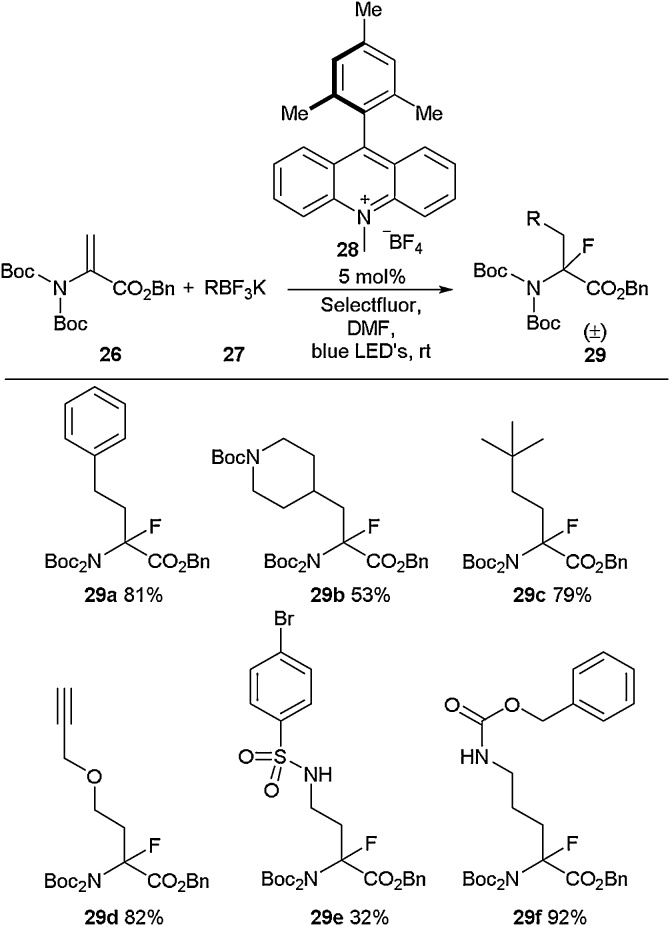
Synthesis of α-fluoro-α-amino acid derivatives via photoredox-catalyzed carbofluorination, Mollander and co-workers [43].

A range of transition metals, beyond palladium, have also been employed to access fluorinated amino acid scaffolds. For example, Liu and co-workers deployed a copper catalyst in their synthesis of aromatic quaternary fluorinated amino acids [42]. It was found that through the coordinating ability of copper ions in tandem with Selectfluor, fluorination could be carried out selectively to enable access to quaternary fluorinated centres (Scheme 8). It was reported that the copper atom afforded selectivity due to its chelation with the pyridine and amide moieties, which directed the Selectfluor reagent. This Cu(II)-mediated catalytic C(sp^3^)-H direct fluorination was employed to access both aliphatic and aromatic amino acids in good to excellent yields. For example, the di-fluorinated compounds **25b** and **25c** were obtained in 75 % and 78 % yields respectively (Scheme 9). Most methodologies for accessing fluorinated aromatic amino acids tend to target installing fluorine-containing groups onto the aromatic rings themselves. In this case, the fluorine atom is installed adjacent to the aromatic system.

Another way to install fluorine atoms into quaternary positions was developed by Mollander and co-workers who employed a catalytic photoredox carbofluorination approach (Scheme 9) [43]. This reaction involved a radical conjugate addition to a protected dehydroalanine followed by subsequent fluorination of the newly generated radical to install a quaternary fluorine. Selectfluor was identified as the fluorination reagent of choice in this reaction, allowing a wide range of fluorinated amino acids to be prepared in good to excellent yields (Scheme 9).

**Scheme 10:**
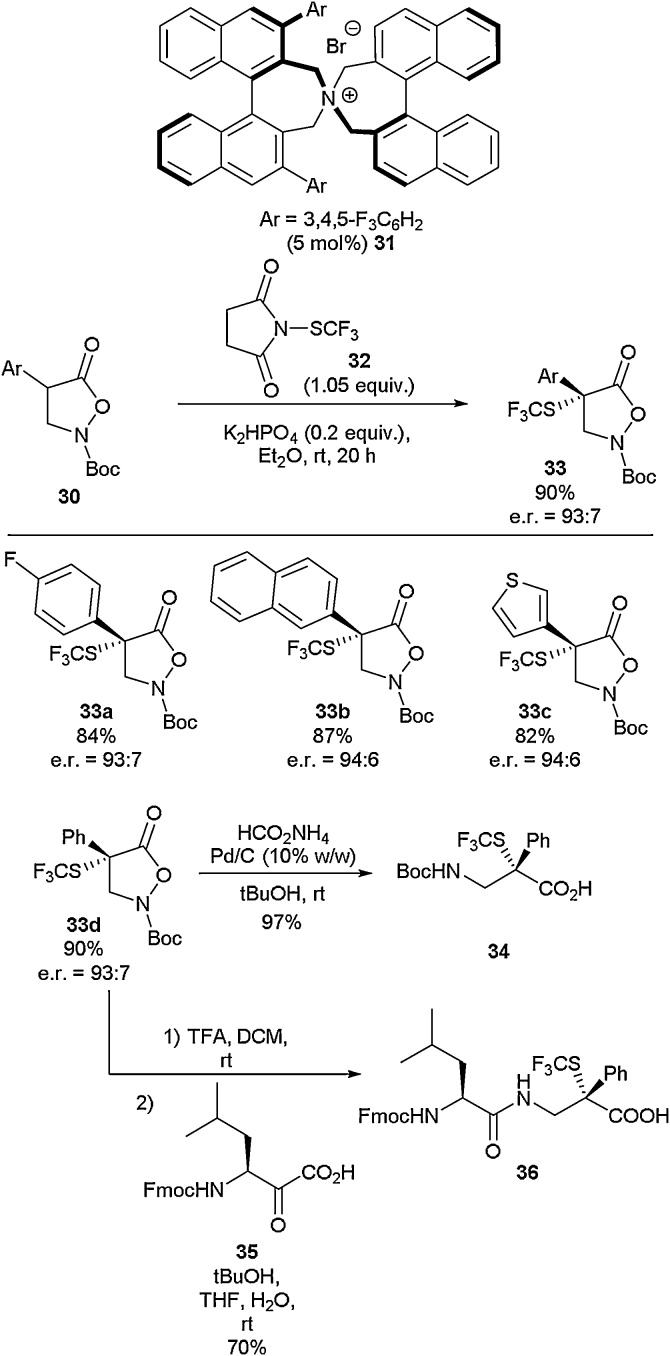
Synthesis of α-aryl-α-SCF_3_-β^2,2^-amino acids, Waser and co-workers [44].

In 2020, Waser and co-workers disclosed the synthesis of α-aryl-α-SCF_3_-β^2,2^-amino acids using an enantioselective, ammonium salt-catalysed α-trifluoromethylthiolation of isoxazolidin-5-ones (Scheme 10) [44]. Starting from compound **30**, the use of catalyst **31** developed by Maruoka [45] along with CF_3_S-transfer reagent **32** allowed isolation of the quaternary substituted species **33d** in excellent yield (90 %) with high enantioselectivity (*e.r*. = 93:7). A range of substituted isoxazolidin-5-ones (**33a-33c**) were found to be amenable to the developed methodology and these compounds were recovered in good to excellent yields, all with high enantioselectivities. It was demonstrated that the generated compounds could be synthetically modified further with relative ease. For example, **33d** was successfully ring-opened to give amino acid **34** in 97 % yield *via* hydrogenation. Compound **33d** could also be deployed directly in peptide synthesis through initial Boc deprotection followed by α-ketoacid-hydroxylamine (KAHA) ligation, affording dipeptide **36** in 70 % yield.

### 2.3 Heterocyclic fluorinated amino acids

Several researchers have developed synthetic methodologies to access aromatic heterocyclic amino acids. Heterocyclic motifs are ubiquitous in natural products and pharmaceuticals, and thus finding methods to access amino acid derivatives is of significant importance to a wide variety of research areas. One class of heterocycle that has seen much attention is the triazolic class [[46], [47], [48], [49], [50]]. Due to the prevalence of click chemistry as a chemical linkage platform, a variety of triazolic amino acids have been developed including fluorinated examples.

**Scheme 11:**
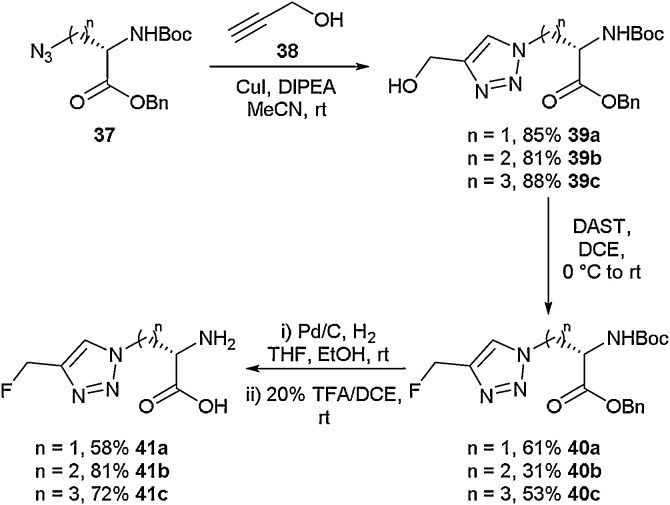
Synthesis of fluorinated triazolic amino acids, Jin and co-workers [51].

Jin and co-workers developed a route to fluorine-containing triazolic amino acids (Scheme 11) [51]. Starting from azide-containing protected amino acid (**37**), a copper-catalysed azide-alkyne cycloaddition (CuAAC) with propargyl alcohol (**38**) was carried out to give the hydroxyl-appended triazoles **39a**-**39c**. This reaction gave the required functionality for deoxyfluorination to be employed using DAST, affording the fluorine-containing amino acids **40a**-**40c**. These were subsequently deprotected to give the free amino acids **41a**-**41c**. In the same report, Jin also outlined the synthesis of an ^18^F radiolabelled version of amino acid **41a**.

**Scheme 12:**
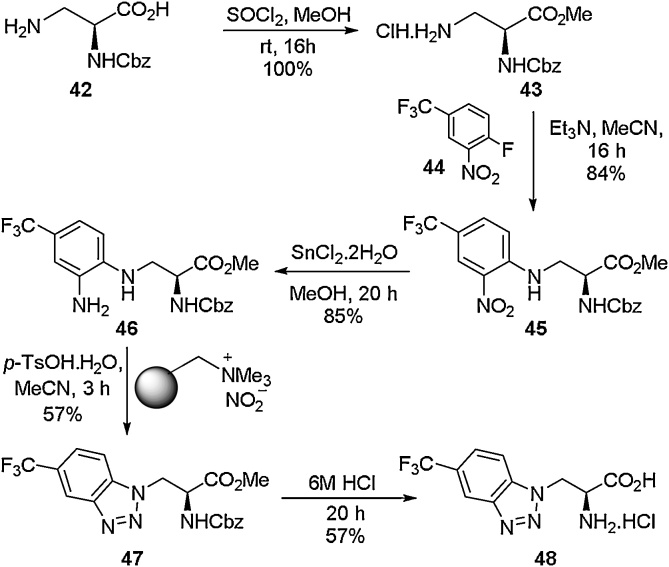
Synthesis of benzotriazole amino acids, Sutherland and co-workers [52].

The synthesis of fluorinated benzotriazole amino acids was reported by Sutherland and co-workers in 2019 (Scheme 12) [52], with benzo-fused amino acids being used for a variety of applications including biological probes and fluorescence imaging access [53,54]. Using an S*_N_*Ar approach, amine **43** was treated with 4-fluoro-3-nitrobenzotrifluoride **44** in the presence of base to give the intermediate **45**. Reduction of the nitro group followed by triazole formation gave the protected trifluoromethyl benzotriazole amino acid **47**, which was then deprotected under acidic conditions to give the free amino acid **48**.

Coumarin-containing amino acids are valuable targets as the coumarin moiety allows them to be utilised as fluorescent probes [[55], [56], [57], [58]]. Being able to access amino acids that combine the fluorescent properties of coumarins with the spectroscopic and physical properties of fluorine is a particularly attractive proposal.

**Scheme 13:**
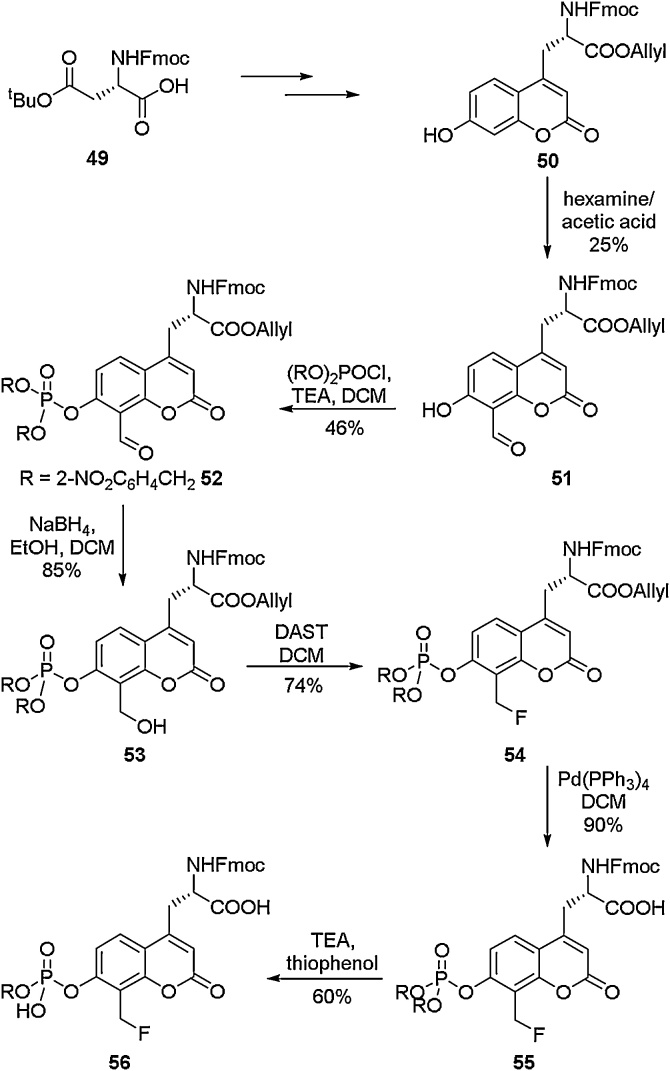
Synthesis of coumarin-containing amino acids, Yao and co-workers [59].

Several routes to fluorine-containing coumarin amino acids have been disclosed. Yao and co-workers developed a route to a phosphotyrosine mimic that included a fluorine-appended coumarin (**56**) (Scheme 13) [59]. They developed this amino acid to be used in a range of bioimaging studies. To access the amino acid, a deoxyfluorination was used as a key step. A hydroxyl group was appended to the coumarin scaffold and subsequently treated with DAST to access the protected amino acid **54**. This compound was then deprotected to give the free amino acid **56** which could be used in SPPS coupling procedures.

Highly fluorinated aromatic species have unique properties. Due to their highly electron deficient nature, perfluoroaromatics can have significant effects on the electronics of molecular systems [60]. Perfluoroaromatic moieties have therefore seen use in materials [61], catalysis [62,63] and organic synthesis [64,65], and have been exploited in peptide [66,67] and peptoid chemistry [68]. Access to highly fluorinated amino acids is therefore an area of much research, with several reports detailing the synthesis of aromatic perfluorinated amino acids.

**Scheme 14:**
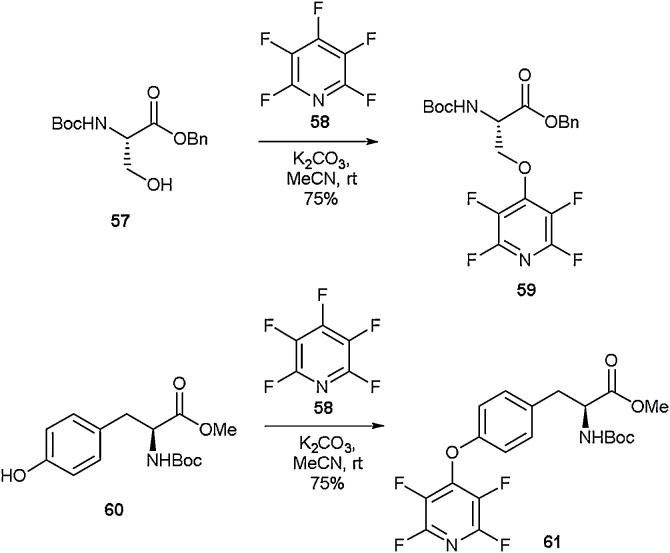
Synthesis of tetrafluoropyridyl amino acids, Cobb and co-workers [70,72].

One strategy that has been developed involves modification of naturally-occurring amino acids with perfluoroaromatic moieties which are prone to S*_N_*Ar reactions. This approach is complementary to methods developed using Mitsunobu chemistry to incorporate fluoroaryl groups onto aliphatic amino acids [69]. Cobb and co-workers have looked to explore this approach across a series of different nucleophilic amino acids (Scheme 14). Modification of serine with pentafluoropyridine (PFP) was shown to occur readily under basic conditions to give amino acid **59** (Scheme 14, top) [70]. This amino acid was demonstrated to be compatible with commonly employed amide bond-forming reagents and was successfully incorporated into a tripeptide. In a further study, it was found that these motifs can undergo elimination to access dehydro-amino acids under mild conditions [71]. Using the same S*_N_*Ar approach, the synthesis of tetrafluoropyridyl(TFP)-tyrosine **61** was reported by Brittain and Cobb (Scheme 14, bottom) [72]. This amino acid was shown to be readily incorporated into peptides, and further it was disclosed that through the addition of a thiol and a fluoride source, the TFP group could be easily removed to give free phenolic tyrosine. The TFP ring was also shown to have profound electronic implications [72,73].

**Scheme 15:**
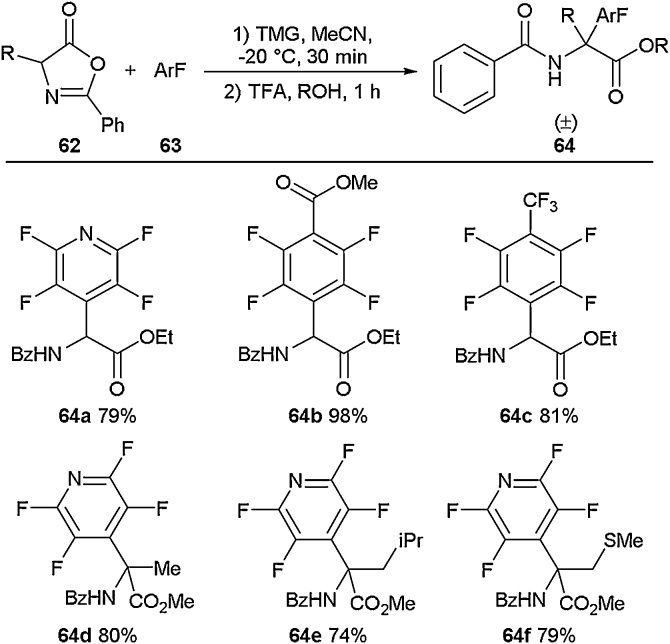
Synthesis of perfluoro-amino acids, Weaver and co-workers [74].

Another method for the incorporation of perfluoroaromatic residues into amino acids was reported by Weaver and co-workers (Scheme 15) [74]. In this report, a series of perfluoroaromatic groups was added directly to the α carbon, removing the need for an existing nucleophilic group. To access these motifs, an oxazolone (**62**) was polyfluoroarylated using a range of perfluoroaromatics (**63**). This reaction was followed by ring-opening and esterification to give a series of protected amino acids (**64a**-**64f**) in good to excellent yields. It was also found that fully substituted amino acids could also be furnished using this synthetic approach.

**Scheme 16:**
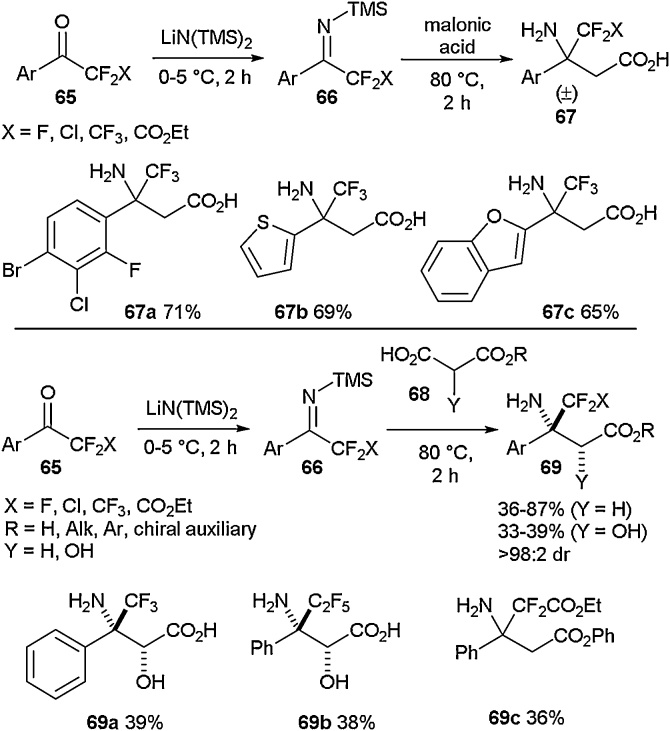
Synthesis of β-fluoroalkyl β-amino acids, Gillaizeau and co-workers [75].

The synthesis of β-fluoroalkyl β-amino acids was reported by Gillaizeau and co-workers in 2019 (Scheme 16) [75]. They employed lithium hexamethyldisilazide and a selection of arylfluoroalkyl ketones (**65**) to generate NH-ketimine intermediates (**66**) that could then undergo decarboxylative Mannich-type reactions. This process allowed access to a variety of both β-aryl-β-fluoroalkyl β-amino acids (**67a-67c**) and α-hydroxy derivatives (**69a-69c**) in good yields. The developed methodology gave high diastereomeric ratios in the generated fluorinated amino acids, and the reactions could be carried out without the need for chromatographic purification. These amino acids were further demonstrated to be applicable in the synthesis of novel heteroaromatic systems [72].

### 2.4 Spirocyclic fluorinated amino acids

**Scheme 17:**
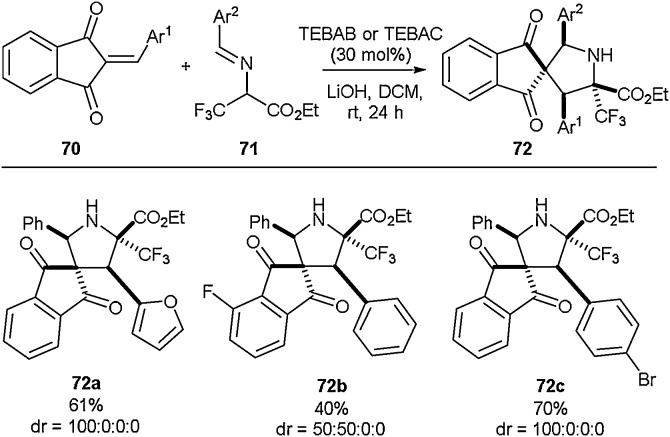
Synthesis of spirocyclic fluorinated amino acids, Waser and co-workers [81].

The preparation of spirocyclic compounds has garnered considerable interest in recent years as these motifs have gained greater significance as medicinally relevant scaffolds (Scheme 17) [[76], [77], [78], [79], [80]]. Waser and co-workers disclosed the synthesis of spirocyclic fluorinated proline derivatives in 2019 [81]. To achieve this, a (3 + 2)-cyclisation approach under phase transfer conditions was implemented. It was found that when using a benzyltriethylammonium bromide (TEBAB) phase transfer catalyst and lithium hydroxide as a base, up to quantitative conversion and complete diastereoselectivity could be achieved in the cycloaddition of the trifluoro-pyruvate imine (**71**) and the benzylidene indandione (**70**). Through variation in the two cycloaddition partners, Waser and co-workers were able to access a variety of multiply substituted spiro-proline derivatives (**72a-72c**) in good to excellent yields. A range of aryl- and heteroaryl-substituents at Ar^1^ and Ar^2^ was successfully installed and the introduction of substituents onto the benzylidene indandione was also achieved. For example, an additional fluorine was successfully incorporated to give compound **72b** in 40 % yield.

## 3 Complex fluorine-containing non-aromatic amino acids

The majority of naturally occurring amino acids are non-aromatic. Given this, a plethora of research has been targeted towards developing routes to access novel aliphatic amino acids. The incorporation of unnatural aliphatic amino acids into peptides and proteins has been demonstrated to modulate a range of physical and chemical properties. Within this area, the ability to introduce fluorine-containing moieties has seen much attention. In this section, approaches for accessing non-aromatic, non-proteogenic complex amino acids will be reviewed.

### 3.1 Cyclic non-aromatic fluorinated amino acids

**Scheme 18:**
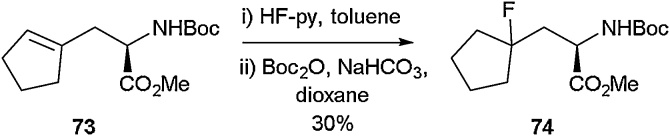
Fluorinated cyclopentane amino acid, Jackson and co-workers [82].

**Scheme 19:**
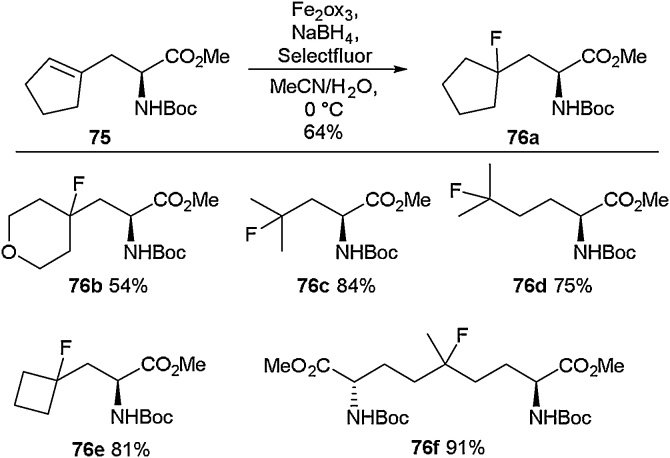
Radical functionalisation of amino acids, Jackson and co-workers [83].

As part of the Jackson group’s studies into the use of Negishi cross-coupling reactions to access unnatural amino acids (detailed above), they synthesised a range of cycloalkenyl derivatives [82]. It was found that treating the cyclopentene containing amino acid **73** with HF.pyridine led to the successful formation of the fluorine-containing amino acid **74** (Scheme 18). The Jackson group would build upon this work, and in 2019 they showed that through the use of radical functionalisation, fluoro, azido and hydroxyl moieties could be installed across olefins to access unnatural amino acids (Scheme 19) [83]. Using a combination of an Fe(III) source with NaBH_4_ and Selectfluor, fluorine atoms were successfully added across a range of cyclic and linear alkenes (**75**). This methodology granted access to a variety of quaternary fluorinated amino acids (**76a-76f**) in good to excellent yields.

**Scheme 20:**
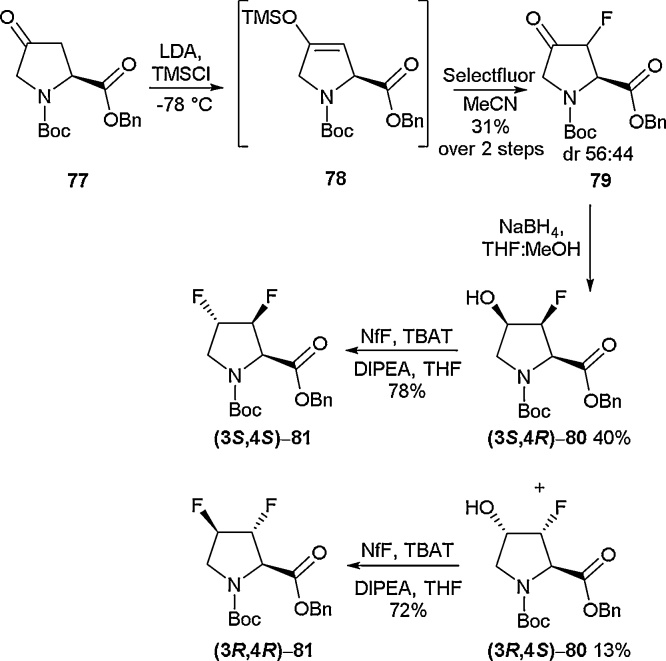
Synthesis of difluoroprolines by electrophilic fluorination, Linclau and co-workers [87].

Proline is often a critical residue in terms of defining a peptide or protein’s secondary structures and it is ubiquitous within the sequences of cyclic peptides. Given this, there has been significant effort invested into accessing fluorinated proline derivatives [84], and multiple reports on the stereoselective synthesis of mono-fluoro prolines have been disclosed [85,86]. Several research groups, however, have focused on the synthesis of prolines substituted with multiple fluorines. It is known that the introduction of a fluorine atom to either the 3 or 4 position of the proline ring can have a dramatic impact on the conformation adopted by the ring. In particular, their presence will encourage puckering of the ring and a change in *cis/trans* isomerisation [85,86]. Having a fluorine atom on both the 3 and 4 positions of the ring leads to a question as to what conformation the different stereoisomers will adopt. In 2019, Linclau and co-workers disclosed an elegant synthesis of 3,4-difluoroprolines and reported for the first time the structural properties of the (3*S*,4*S*) and (3*R*,4*R*) stereoisomers (Scheme 20) [87]. In order to access the (3*S*,4*S*) and (3*R*,4*R*) stereoisomers, an electrophilic fluorination approach was employed. From ketoproline **77**, the silyl enol ether (**78**) was formed by treatment with LDA and TMSCl. Next, electrophilic fluorination was conducted using Selectfluor to give fluoro-keto-proline **79** in 31 % yield over two steps. Reduction of the ketone to the alcohol gave the two stereoisomers **(3*S*,4*R*)-80** and **(3*R*,4*S*)-80**, which were readily separated. These hydroxyl-fluoro-prolines had been reported previously by Ciulli and co-workers in 2018 [88]. Further exposure to deoxyfluorination conditions afforded the two difluoroprolines **(3*S*,4*S*)-81** and **(3*R*,4*R*)-81** in 78 % and 72 % yields respectively.

**Scheme 21:**
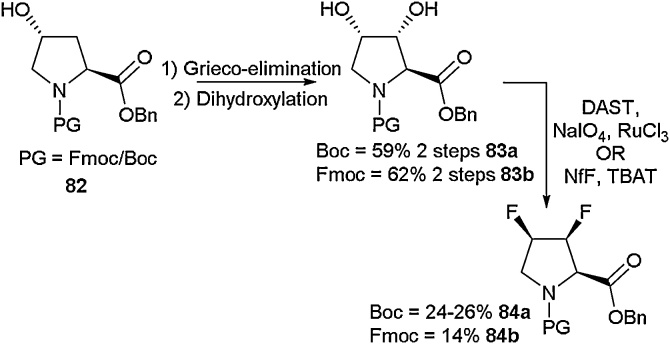
Grieco-elimination approach to difluoroprolines, Linclau and co-workers [87].

In order to access the (3*S*,4*R*) and (3*R*,4*S*) stereoisomers, Linclau and co-workers took a different approach (Scheme 21). Using a Grieco-elimination followed by dihydroxylation, the diols (**83a-83b**) were isolated. This material could then undergo deoxyfluorination to give the desired difluoroprolines **84a-84b** in moderate yield.

**Scheme 22:**
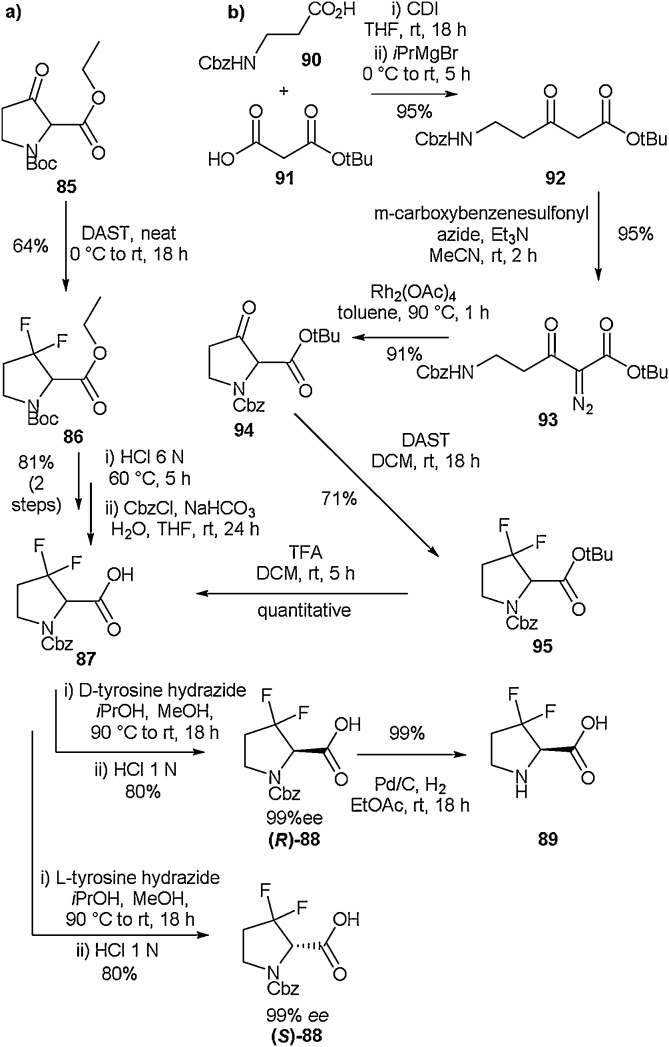
a) Synthesis of 3,3-difluoroproline on small scale b) Synthesis of 3,3-difluoroproline on multigram scale, Kamenecka and co-workers [89].

The multigram-scale synthesis of enantiopure 3,3-difluoroproline was reported by Kamenecka and co-workers in 2016 (Scheme 22) [89]. The method was adjusted slightly depending on the reaction scale (Scheme 22a vs b), and different protecting group strategies could be employed as required. In order to prepare several grams of **89** (Scheme 22a), commercially available ethyl *N*-Boc-3-oxopyrrolidine-2-carboxylate (**85**) was fluorinated with DAST in the absence of solvent, giving a racemic mixture of **86** in a yield of 64 % on a 5 g scale. Further exposure to 6 N HCl at 60 °C for 5 h resulted in simultaneous Boc and ethyl ester deprotection. Cbz protection of the amine was then employed in order to allow for chiral resolution with D- or L-tyrosine hydrazide according to established literature procedures, [90,91], yielding **(*R*)-88** or (S)-**88** respectively (99 % *ee*). A final Cbz deprotection step afforded enantiopure 3,3-difluoroproline (**89**) as desired.

When larger scale synthesis of 3,3-difluoroproline was required (>30 g), a slightly different approach was taken (Scheme 22b). The cyclic α-amino-β-keto ester scaffold **94** was first built according to a modified procedure described by Moyer et al. in 1985 [92]. This consisted of a Masamune-Claisen condensation followed by a diazo transfer in order to generate **93**. Cyclisation to give **94** could then be achieved in the presence of Rh_2_(OAc)_4_. Additional steps including deoxofluorination were then carried out under DAST, giving racemic compound **87** which underwent chiral resolution as before.

**Scheme 23:**
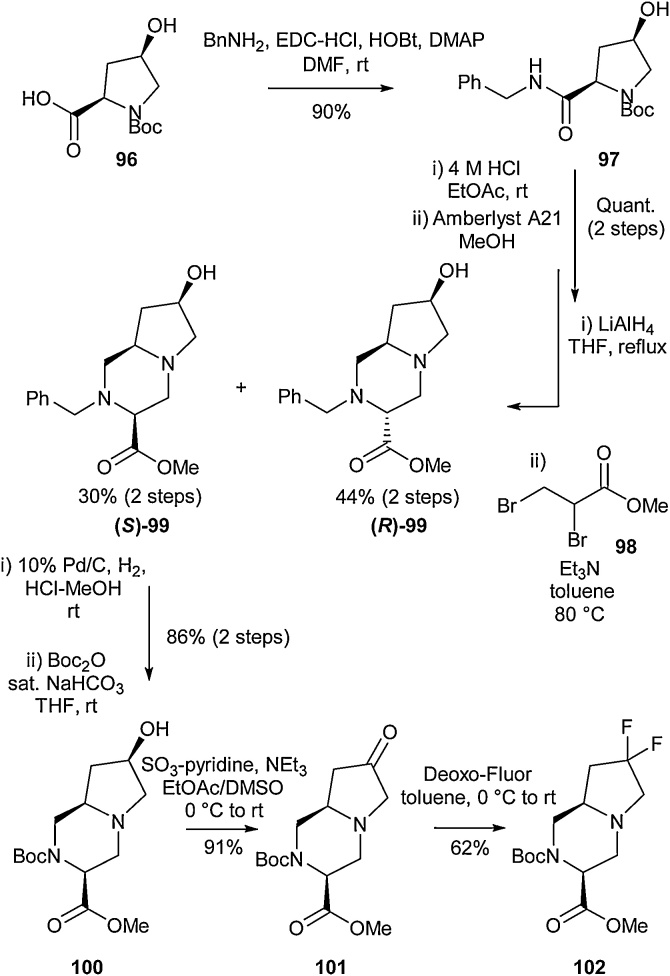
Synthesis of 7,7-difluorooctahydropyrrolo[1,2-*a*]pyrazine derivatives, Ishikawa and co-workers [93].

In 2013, Ishikawa and co-workers reported synthetic methodology that allowed for successful isolation of the 7,7-difluorooctahydropyrrolo[1,2-*a*]pyrazine derivative **102** (Scheme 23) [93]. Initial condensation of the commercially available (4R)-hydroxyproline derivative **96** and benzylamine using known conditions led to the successful formation of amide **97**. Further Boc deprotection and subsequent reduction with lithium aluminium hydride gave the diamine species. This compound could then be cyclised with methyl 2,3-dibromopropionate under basic conditions to afford the octahydropyrrolo[1,2-*a*]pyrazine scaffold **99** according to a method adapted from a previously reported protocol [94]. Separation of **(*R*)-99** and **(*S*)-99** was possible by silica gel column chromatography, allowing the desired (7*R*)-hydroxy derivative ((S)-**99**) to be isolated. Hydrogenation of the *N-*benzyl functionality was then followed by Boc protection, leading to **100**. The hydroxyl group could then be oxidised to the ketone before being treated with Deoxo-Fluor, yielding 7,7-difluoro derivative **102** (Scheme 23).

**Scheme 24:**
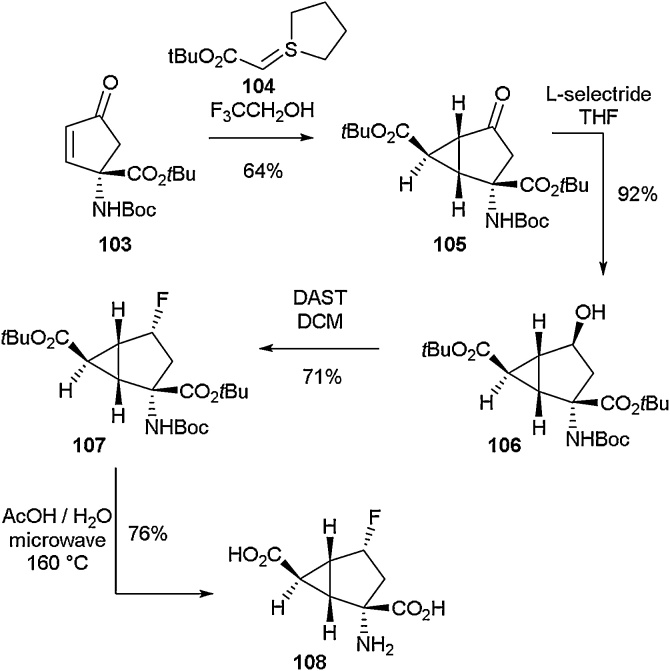
Synthesis of 4-fluoro-2-aminobicyclo[3.1.0]hexane-2,6-dicarboxylate, Monn and co-workers [95].

The synthesis of 4-fluoro-2-aminobicyclo[3.1.0]hexane-2,6-dicarboxylate **108** was reported by Monn and co-workers in 2013 for the purpose of carrying out biological studies (Scheme 24) [95]. Initial reduction of ketone **105**, a versatile intermediate prepared from enone **103** [96], resulted in the formation of β-carbinol **106** [97]. Further treatment with DAST allowed successful installation of a fluorine atom on the α-face of the bicyclohexane structure at the C4 position (**107**). Full deprotection through microwave-assisted solvolysis gave a zwitterionic form of **108** in a yield of 76 % [98]. Various attempts to synthesise the β-fluoro analogue proved largely unsuccessful as only trace amounts of the desired compound were salvaged from the crude material.

**Scheme 25:**
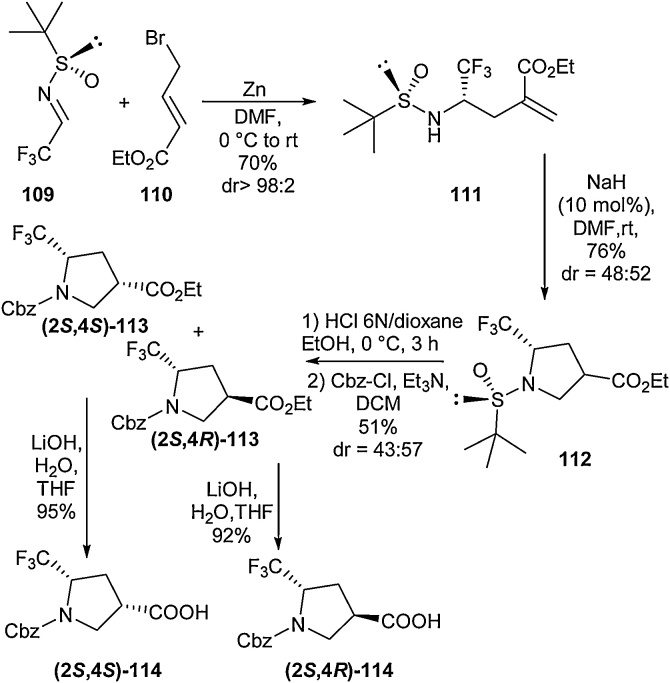
Access to trifluoromethyl β-proline derivatives, Hao et al. [101].

Trifluoromethylation of prolines is another means by which fluorine atoms can be introduced to modulate the residue’s electronic and physical properties. Researchers have targeted the installation of the CF_3_ and carboxylic acid moieties onto different positions to decorate the proline ring [99,100]. In 2018, Hao et al. disclosed the synthesis of novel trifluoromethyl β-proline derivatives (Scheme 25) [101]. In order to access these scaffolds, an intramolecular 5-*endo*-trig cyclisation of a sulfinylamine (**111**) was targeted as a key step. Under basic conditions, this cyclisation occurred readily to give the protected β-proline derivative **112** in 76 % yield. This compound could then be treated with 6 N HCl in dioxane to remove the sulfinyl group to give the protected amino acids **(2*S*,4*S*)-113** and **(2*S*,4*R*)-113**. Finally saponification was conducted to give the amino acids **(2*S*,4*S*)-114** and **(2*S*,4*R*)-114**.

**Scheme 26:**
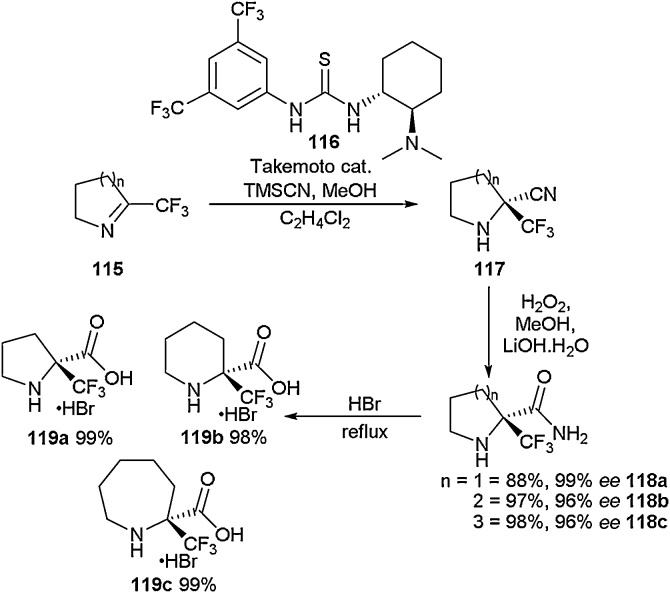
Synthesis of cyclic α-perfluoroalkylated amino acids, Nenajdenko and co-workers [102].

Nenajdenko and co-workers studied the enantioselective synthesis of α-perfluoroalkylated prolines (including trifluoromethyl) and also generated 6- and 7-membered homologues (Scheme 26) [102]. To achieve this, a stereoselective Strecker reaction was employed using a catalyst developed by Takemoto and co-workers [103], forming nitrile **117** in 95 % *ee*. Treatment of the nitrile (**117**) with hydrogen peroxide and lithium hydroxide in methanol followed by exposure to HBr led to the successful synthesis of enantiopure amino acids **119a-119c**.

**Scheme 27:**
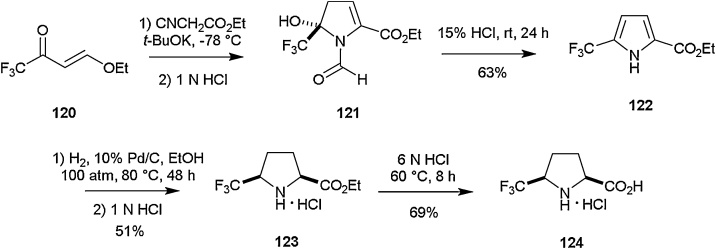
Synthesis of 5-trifluoromethyl proline, Haufe and co-workers [106].

The successful incorporation of CF_3_ groups into α-prolines has been reported by various groups including Brigaud and co-workers [104] and Ulrich et al. [105]. One particular example describes the synthesis of 5-trifluoromethyl proline **124** by Haufe and co-workers in 2012 according to Scheme 27 [106]. Starting from trifluoromethyl enone **120**, reaction with ethyl isocyanoacetate and potassium *tert*-butoxide enabled isolation of **121** as the major tautomer (85 %) in CDCl_3_ (minor tautomers not shown in scheme). Subsequent exposure to acidic conditions brought about elimination of formic acid, allowing generation of pyrrole **122** in a yield of 63 %. Further catalytic hydrogenation to **123** was achieved through the employment of conditions already reported in the literature [107,108], followed by hydrolysis to acid **124** in the presence of 6 N HCl.

**Scheme 28:**
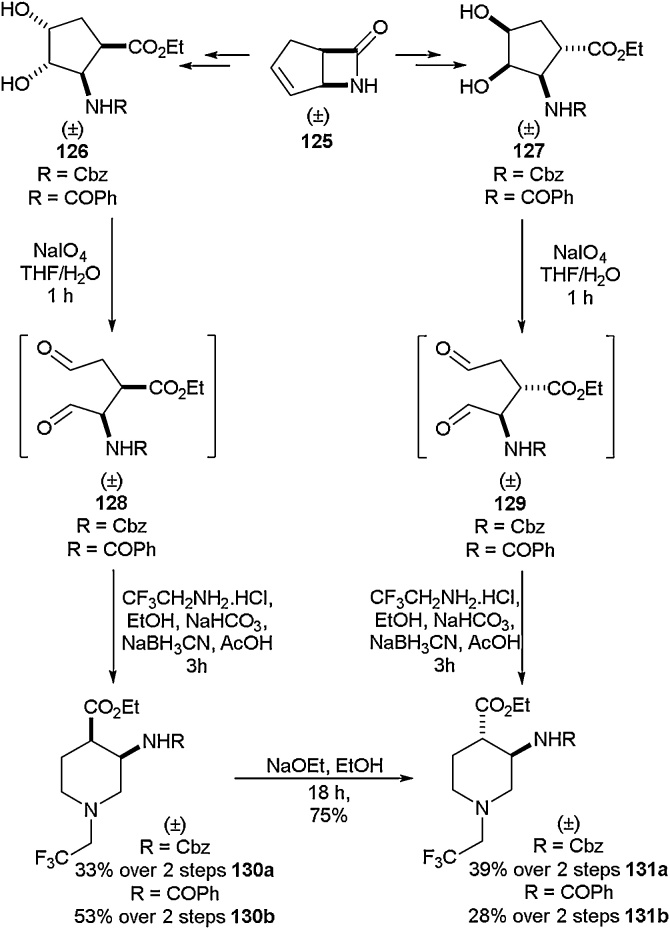
Synthesis of fluorinated piperidine amino acids, Fülöp and co-workers [112].

The synthesis of fluorinated piperidine, azepane and bicyclic β-amino acids is an area in which several studies have been conducted [[109], [110], [111]], with one such study being disclosed by Fülöp and co-workers in 2016 [112]. In order to achieve the desired target, it was envisaged that subjecting a commercially available trifluoromethylated bicyclic β-lactam to ring opening using oxidative cleavage, followed by ring closure and expansion by reductive amination, would give the expanded protected amino acids (Scheme 28). Starting from lactam **125**, ethanolysis followed by *N*-protection and cis-dihydroxylation gave the cis-amino esters **126** and **127**. Oxidative ring cleavage gave the opened diformylamino esters **128** and **129,** which were found to be unstable. These compounds were used without isolation in the reductive amination with 2,2,2-trifluoroethylamine to give the six-membered piperidine amino ester derivatives **130a-130b** and **131a-131b**. A similar strategy was implemented to access the analogous azepane derivatives, starting from a different lactam possessing an extra carbon atom in its rings.

**Scheme 29:**
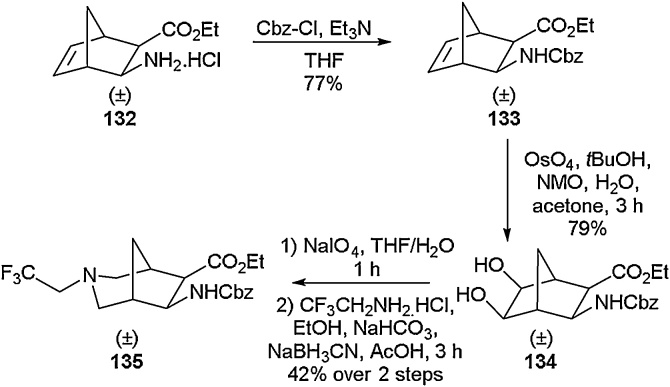
Access to fluorinated bi-cyclic amino acids, Fülöp and co-workers [112].

Within the same report, the isolation of bi-cyclic fluorinated amino acids was also detailed (Scheme 29) using a similar strategy. Bi-cyclic amino acids are an important class of compounds with their 3D structures allowing for interesting applications, especially in biological probes and drug candidates [113,114]. Cbz protection of **132** followed by dihydroxylation gave the cis-diol **134** which could then undergo ring opening, reductive amination and ring closure to generate novel bi-cyclic fluorinated amino acid **135** in good yield.

**Scheme 30:**
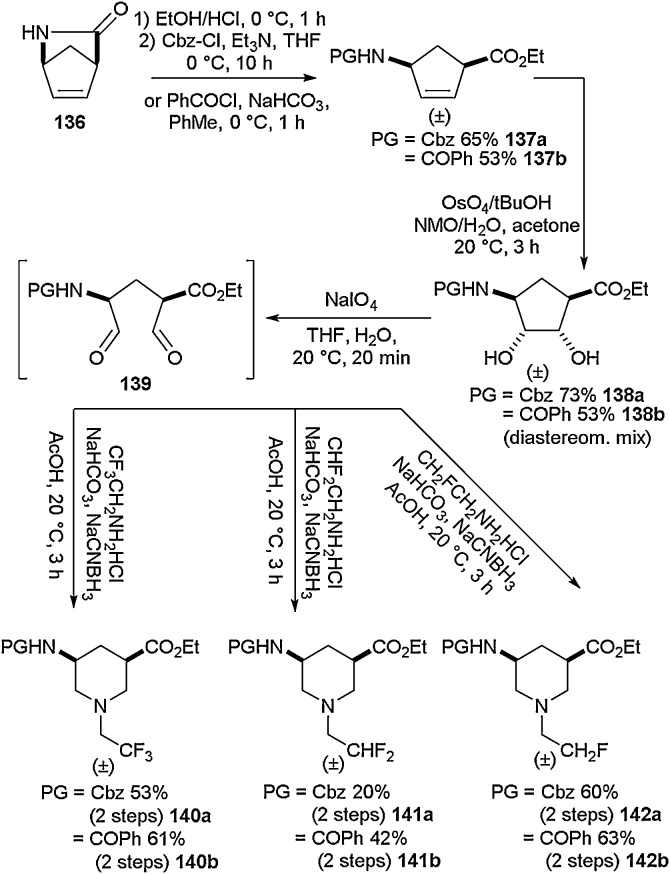
Synthesis of fluorinated γ-amino acids using a ring expansion approach, Kiss and co-workers [116].

Kiss and co-workers would utilise this approach in several other reports [115], including deployment of the same ring expansion strategy to access fluorinated γ-amino acids (Scheme 30) [116] using the readily commercially available Vince lactam **136**. The same approach of dihydroxylation, ringopening and reductive amination was utilised to access a variety of fluorine-containing piperidine γ-amino acids (**140-142**). In addition to the examples shown in Scheme 30, a further range of amines was employed in the reductive amination step in order to access a selection of additional examples. The synthesis of tetrahydropiperidine and lactam-containing products was also detailed in the same report.

**Scheme 31:**
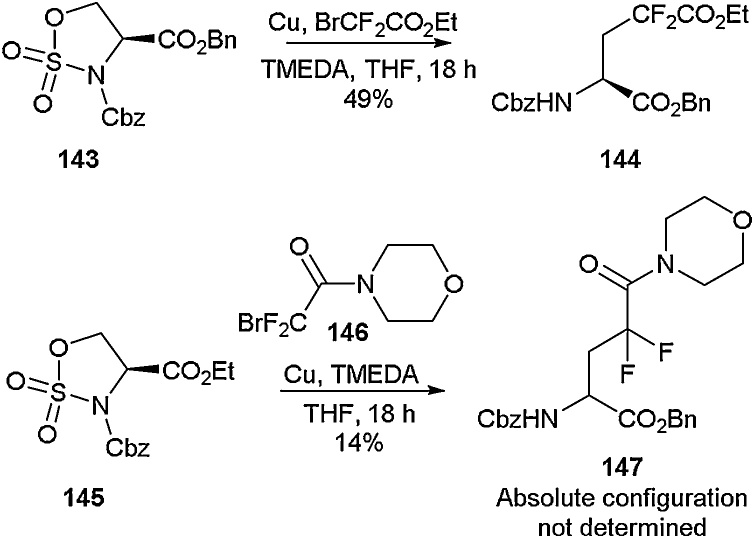
Access to β-fluoroalanines and difluoroglutamic acid derivatives, Bolek and Ignatowska [117].

The application of ring-opening strategies has also been demonstrated in the synthesis of β-fluoroalanines and difluoroglutamic acid derivatives by Bolek and Ignatowska (Scheme 31) [117]. Starting with the cylic sulfamidate **143**, ring-opening was achieved using a mixture of copper, BrCF_2_CO_2_Et and TMEDA. This strategy delivered the 4,4-difluoroglutamic acid derivative **144** in 49 % yield. Using the same approach with *N*-morpholine bromodifluoroacetamide, the novel amino acid **147** was obtained in 14 % yield.

**Scheme 32:**
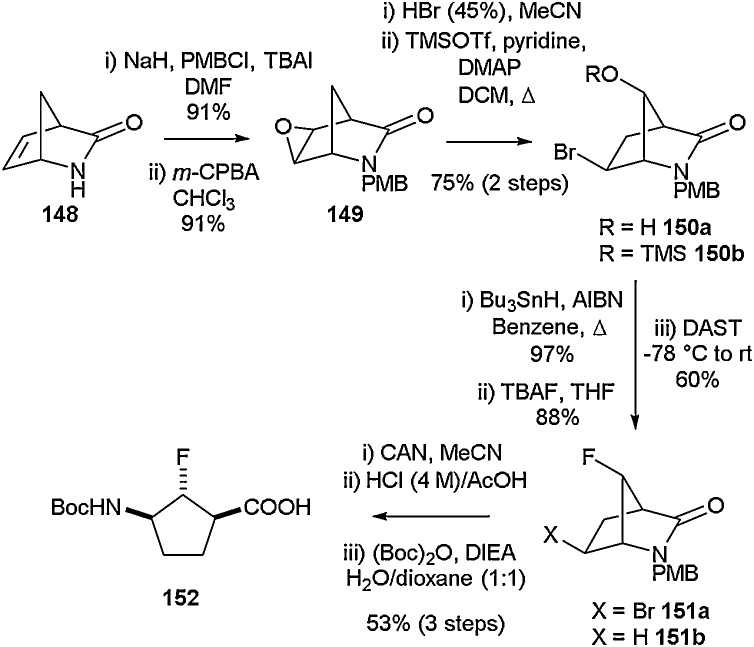
Synthesis of *cis*-fluorinated cyclic γ-amino acids, Granja and co-workers [120].

Cyclopentane-containing amino acids are another class of compounds which have seen increasing interest [118,119]. They are particularly beneficial as they can be introduced in the place of proline to alter physical and chemical properties of compounds. The synthesis of fluorinated cyclic γ-amino acids in which the amino and carboxylic acid groups adopt a *cis* configuration relative to each other was successfully achieved by Granja and co-workers (Scheme 32) in 2013 [120]. This synthesis was accomplished using a procedure similar to that already described in the literature [121]. The first step in the process involves protection of Vince lactam **148** with *p*-methoxybenzylchloride followed by treatment with *m*-CPBA in order to generate epoxide **149**. Epoxide ring opening with hydrogen bromide and subsequent addition of trimethylsilyl triflate afforded **150b**. TMS protection enabled separation of the hydroxy-derivative **150a** from its regioisomer, which was isolated as a by-product in a yield of 12 %. Further to this, radical debromination followed by TMS deprotection and the addition of TBAF led to the formation of **151b**. This was then transformed into Boc-2-F-γ-Acp-OH (**152**) through removal of PMB with ceric ammonium nitrate followed by hydrolysis of the lactam and Boc protection of the resulting free amine. This building block could then be carried forward for use in peptide synthesis.

**Scheme 33:**
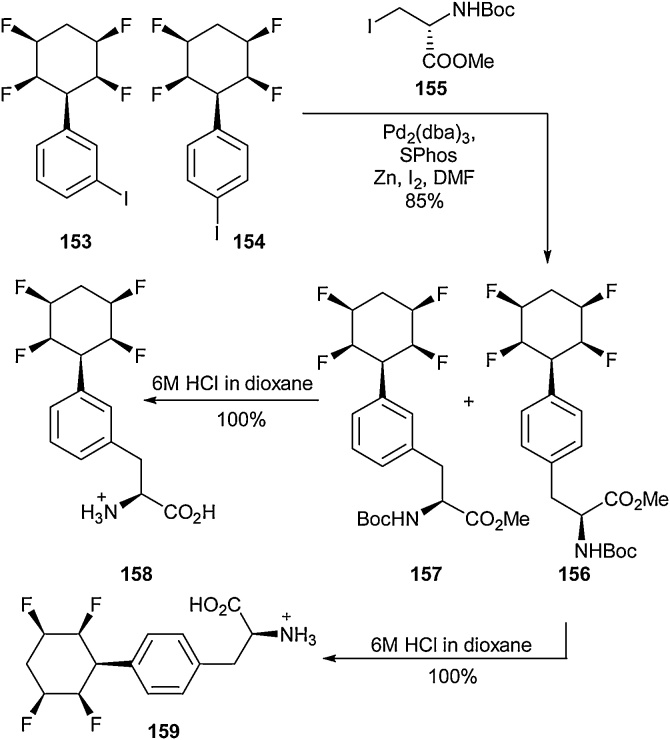
Janus-faced fluorinated cyclohexyl amino acids, O’Hagan and co-workers [124].

The ability to stereoselectively access aliphatic fluorinated amino acids has been studied by the O’Hagan group. The synthesis of cyclohexyl fluorinated amino acids has been explored by others, but these reports focused on singly or doubly fluorinated analogues [122,123] whilst O’Hagan wished to access highly fluorinated cyclohexanes. Specifically, the synthesis of compounds in which all fluorine substituents are installed on a single face of a ring. These Janus-faced molecules are interesting due to the extreme differences in electronegativity from one face of the ring to the other, which leads to large molecular dipole moments and unique polar hydrophobic properties. In 2015, O’Hagan and co-workers disclosed the synthesis of tetrafluorocyclohexyl tyrosine derivatives (Scheme 33) [124]. Using a mixture of regioisomers of the iodo-aryl compounds **153** and **154**, a Negishi cross-coupling approach was employed to access a mixture of protected amino acids (**156** and **157**). These two regioisomers were found to be readily separated by column chromatography and the two compounds were deprotected to give the free amino acids **158** and **159** in quantitative yields. Following their synthesis, both *N-* and *C-*terminal amide coupling was demonstrated to generate short peptides.

**Scheme 34:**
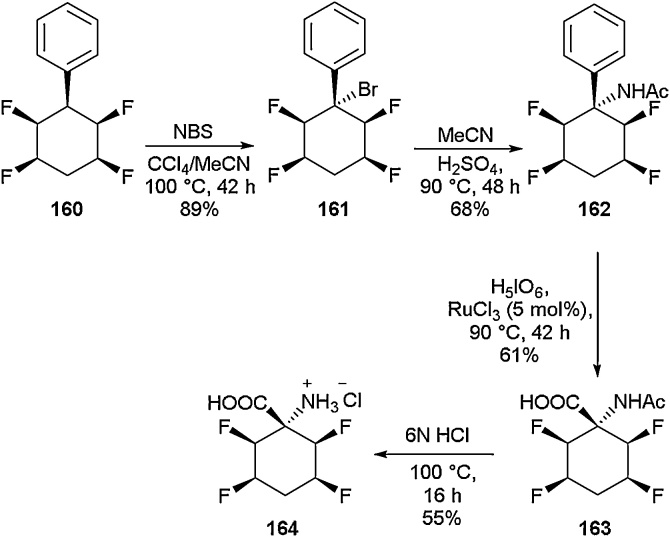
Fluorinated cyclohexyl amino acids, O’Hagan and co-workers [125].

This report was followed up by O’Hagan and co-workers in 2018 where a new family of fluorinated amino acids was developed (Scheme 34) [125]. Starting from the all-*cis* tetrafluorocyclohexane **160** [126], bromination was carried out to give the quaternary substituted brominated compound **161** in 89 % yield. Treating **161** with acetonitrile and sulphuric acid under Ritter conditions gave the acetamide **162** in 68 % yield. Under biphasic conditions, oxidative treatment of acetamide **162** with H_5_IO_6_ and RuCl_3_ (5 mol%) gave the carboxylic acid derivative **163** in a yield of 61 %. This could then be deprotected under acidic conditions to give the free amino acid **164** in 55 % yield. In addition to the amino acid derivative **164,** a range of other highly fluorinated species were also reported. This methodology was found to be applicable to the synthesis of quaternary amines and also α-fluoroamides.

**Scheme 35:**
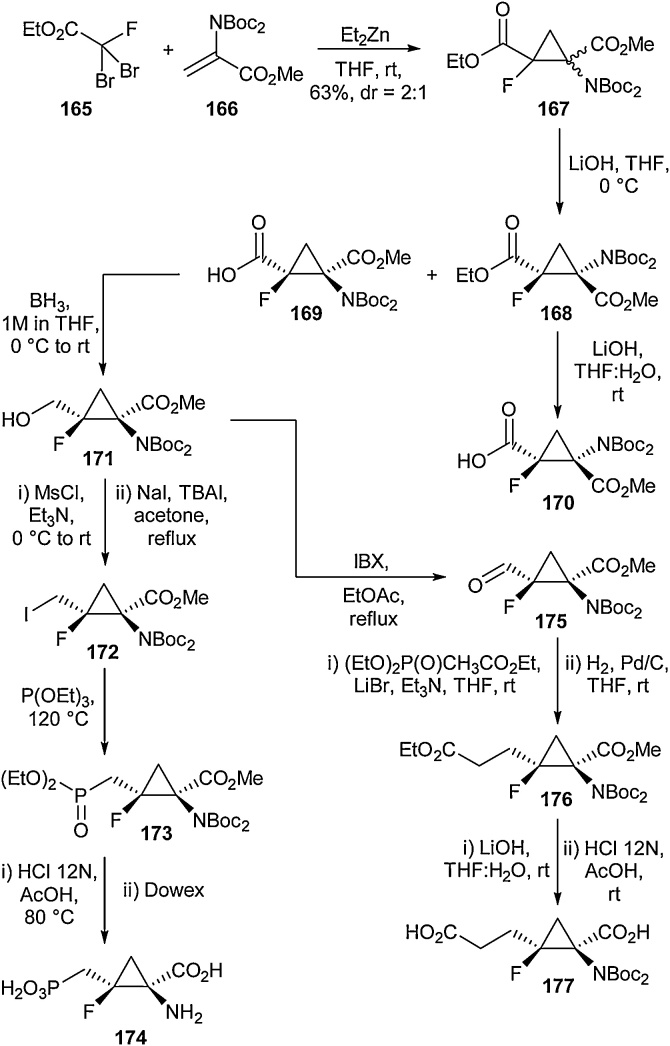
Routes to cyclopropane amino acids, Jubault and co-workers [127].

The synthesis of α-amino-β-fluorocyclopropanecarboxylic acids was disclosed by Jubault and co-workers in 2012 as part of a study into their agonistic behaviour towards metabotropic glutamate receptor 4 (Scheme 35) [127]. To begin their synthesis, a Michael induced ring closure reaction between the dibromofluoroacetate **165** and the amino acrylate **166** in the presence of Et_2_Zn was carried out to give the fluorocyclopropane **167** in 63 % yield and a *dr* of 2:1 [128]. The ethyl ester could be regio- and diastereoselectively saponified to give a separable mixture of the carboxylic acid **169** and ethyl ester **168**. The ethyl ester could then be saponified at room temperature to access both *Z* and *E* diastereoisomers of the carboxylic acid **169** and **170**. Borane reduction of carboxylic acid **169** gave the alcohol (**171**), which underwent mesylation followed by displacement with sodium iodide to give the iodo compound **172**. An Arbuzov condensation with triethylphosphite gave the phosphonate **173**, which was then deprotected with concentrated HCl to give the amino acid **174** in 21 % overall yield. A dicarboxylic acid was also reported in the same paper. Starting from alcohol **171**, an IBX oxidation gave the aldehyde **175** in quantitative yield. This was then treated under Warner-Wadsworth-Emmons conditions before undergoing hydrogenation in order to generate the ethyl ester (**176**). Subsequent deprotection under acidic conditions afforded the free amino acid **177** in 23 % overall yield. In addition, the researchers also outlined the synthesis of several further derivatives including sulfomethylcyclopropane examples.

**Scheme 36:**
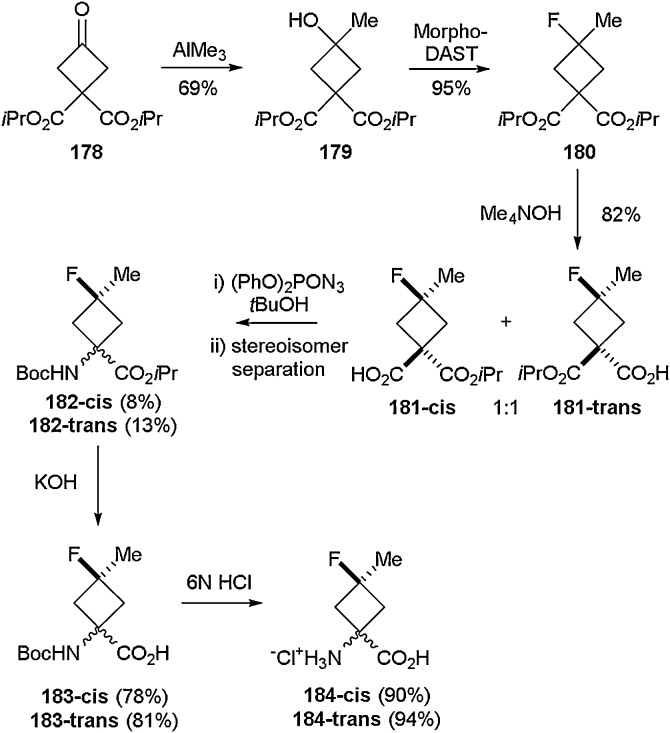
Synthesis of cis‐and trans‐1‐amino‐3‐fluoro‐3‐methylcyclobutanecarboxylic acids by Haufe and co-workers [132].

Stereoselective access to cyclobutane and cyclobutane derived amino acids has been pursued by several groups [[129], [130], [131]]. One example was reported by Haufe and co-workers who disclosed the synthesis of cis‐and trans‐1‐amino‐3‐fluoro‐3‐methylcyclobutanecarboxylic acids in 2016 (Scheme 36) [132]. Starting from the commerically available oxo-ester **178**, methylation using AlCl_3_ was carried out to generate hydroxyl containing **179** in 69 % yield. This compound underwent deoxyfluorination using Morpho-DAST to give racemic fluorinated ester **180**. Hydrolysis of **180** with Me_4_NOH gave a 1:1 mixture of the two stereoisomers **181-cis** and **181-trans** in 82 % yield. Cutis rearrangement followed by separation of stereiomers via fractional crystallisation gave enantiomerically pure samples of **182-cis** and **182-trans** in 8% and 13 % yield respectively. Deprotection of both the ester and Boc group led to the desired stereomerically pure amino acids **184-cis** and **184-trans**. The overall yields for this route were calculated at 3% for **184-cis** and 5% for **184-trans**. The physical chemical properties of these compounds were probed in the same report. Interestingly it was found that the amino groups of the two stereiosomers of **184** displayed different basicities whilst the carboxylic acid moiteies of the same compound had identical basicity profiles.

### 3.2 Acyclic fluorinated non-aromatic amino acids

**Scheme 37:**
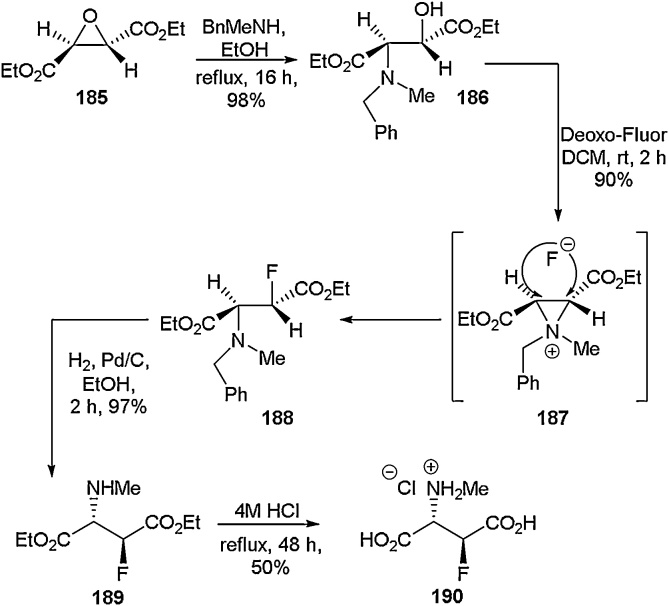
Stereoselective synthesis of 3-fluoro-*N*-methyl-d-aspartic acid, O’Hagan and co-workers [140].

Acyclic non-aromatic motifs are the most prevalent in naturally occurring amino acids. Therefore, the ability to access either fluorinated analogues or completely novel acyclic motifs is of utmost importance for applications such as biological probes and peptide therapeutics [[133], [134], [135], [136], [137], [138], [139]]. There have been many reports of the synthesis of singly fluorinated analogues of naturally occurring acyclic amino acids [12]. One such example describes the stereoselective synthesis of 3-fluoro-*N*-methyl-d-aspartic acid by O’Hagan and co-workers (Scheme 37) [140]. Starting from the epoxy succinic ester **185**, ring opening was achieved using *N*-benzylmethylamine to give compound **186** in 98 % yield. Treatment of the amino alcohol **186** with Deoxo-Fluor led to stereoselective fluorination *via* an aziridinium intermediate **187** to give **188** in 90 % yield. Removal of the benzyl group from the amine nitrogen was accomplished through hydrogenation with Pd/C to give **189** in 97 % yield. Subsequent treatment with HCl gave the fully deprotected amino acid as a single diastereoisomer (**190**) in 50 % yield. In the same report, the other diastereoisomers of **190** were also synthesised starting from an achiral meso-epoxide. Diastereoisomer formation was then followed by chromatographic separation in order to enable isolation of single diastereoisomers. These amino acids were then used to probe their agonist activity at glutamate receptors. It was found that the stereochemistry of the amino acids was vital to their biological activity. For example, the (2*S*,3*S*) analogue had good activity whilst the (2*S*,3*R*) version was almost completely inactive.

**Scheme 38:**
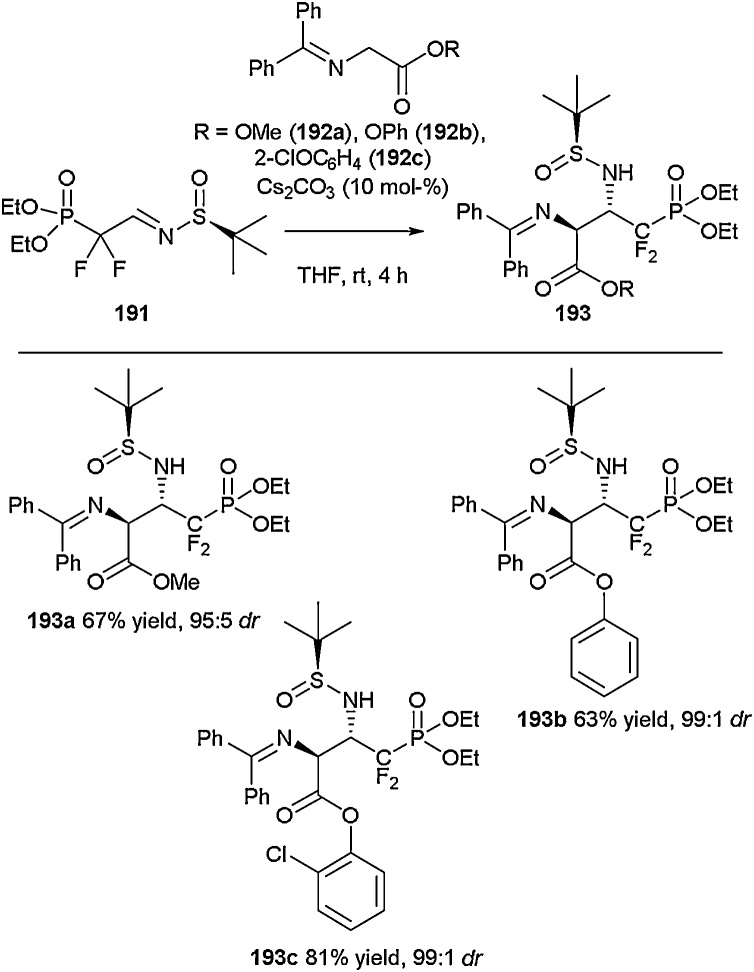
Synthesis of β-amino-γ,γ-difluoro-ω-phosphonoglutamic acid derivatives, Soloshonok and co-workers [141].

The synthesis of β-amino-γ,γ-difluoro-ω-phosphonoglutamic acid derivatives was achieved by Soloshonok and co-workers in 2017 [141] through the utilisation of a base-catalysed Mannich addition involving electrophilic chiral *N-*(*tert*-butylsulfinyl)difluoro(phosphoryl)imine **191** [142] and a nucleophilic glycinate-derived Schiff base (Scheme 38). In fact, the use of *N*-tert-butylsulfinylimines has been utilised by several groups to access acyclic fluorinated amino acids [143]. A selection of Schiff bases including **192a**, **192b** and **192c** was screened in order to determine the optimal conditions with respect to both isolated yield and diastereoselectivity. The imine functionality was subsequently hydrolysed to the free amine and exploited for the synthesis of an all-α-amino acid derived dipeptide according to standard amide bond coupling procedures. Alternatively, tosylation of the α-amino group followed by targeted deprotection of the orthogonal sulfinylamide situated on the β-amino group could be employed in order to allow generation of mixed α,β-amino acid derived dipeptides.

**Scheme 39:**
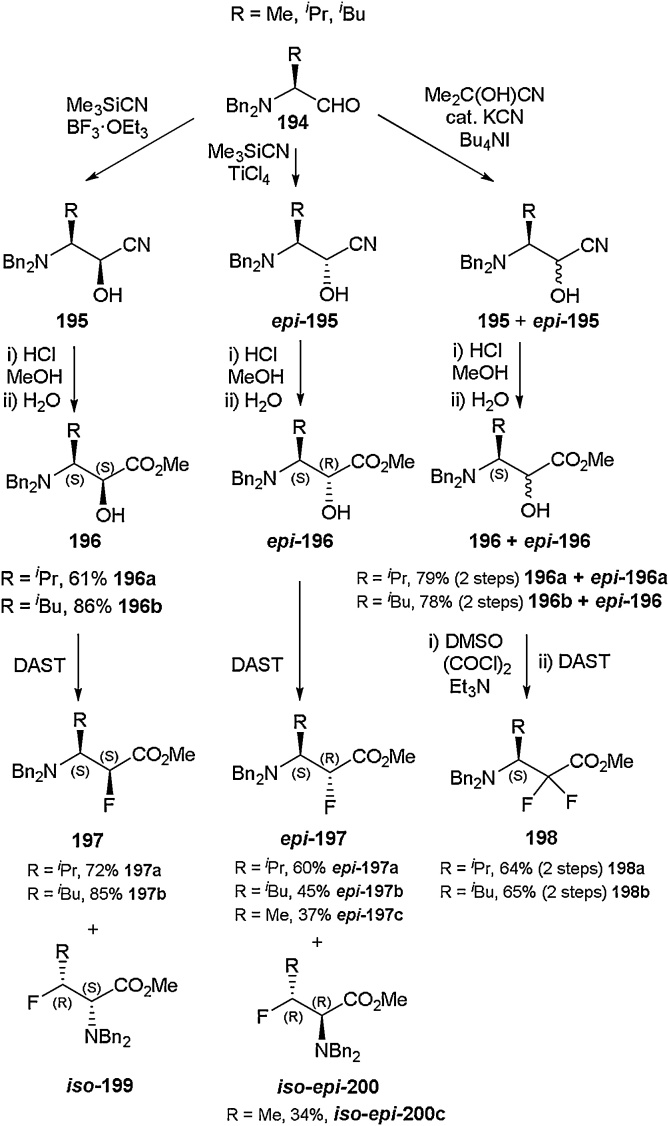
Synthesis of α-fluoro-β-amino acid derivatives, Seebach and co-workers [144].

Seebach and co-workers documented the successful isolation of α-fluoro-β-amino acid derivatives starting from the naturally occurring α-amino acid building blocks alanine, valine, leucine and threonine (Scheme 39) [144]. Aldehyde **194**, possessing variable R groups (Me, *^i^*Pr and *^i^*Bu), was obtained through *N,N*-dibenzylation followed by reduction of the corresponding amino acids [145]. Further exposure to trimethylsilyl cyanide in the presence of either boron trifluoride etherate (non-chelation-controlled) or titanium tetrachloride (chelation-controlled) led to the formation of **195** and ***epi-***195, respectively, according to Reetz’s diastereoselective cyanohydrin reaction [146]. A subsequent Pinner reaction allowed generation of 3-amino-2-hydroxy carboxylic acid esters **196** and ***epi-*196**. Installation of a fluorine atom with DAST led to the isolation of constitutional isomers **197** and **iso-199** as well as ***epi-*197** and **iso*-epi-*200**. Alternative reaction of aldehyde **194** with a non-diastereoselective cyanating agent [147] followed by Swern oxidation and subsequent treatment with DAST could be employed in order to prepare enantiopure geminal difluoro-β-amino acid ester **198**, as illustrated by Scheme 39. Further minor manipulations led to the acquisition of building blocks suitable for incorporation into pyrimidinones as well as cyclic β-tri and β-tetrapeptides.

**Scheme 40:**
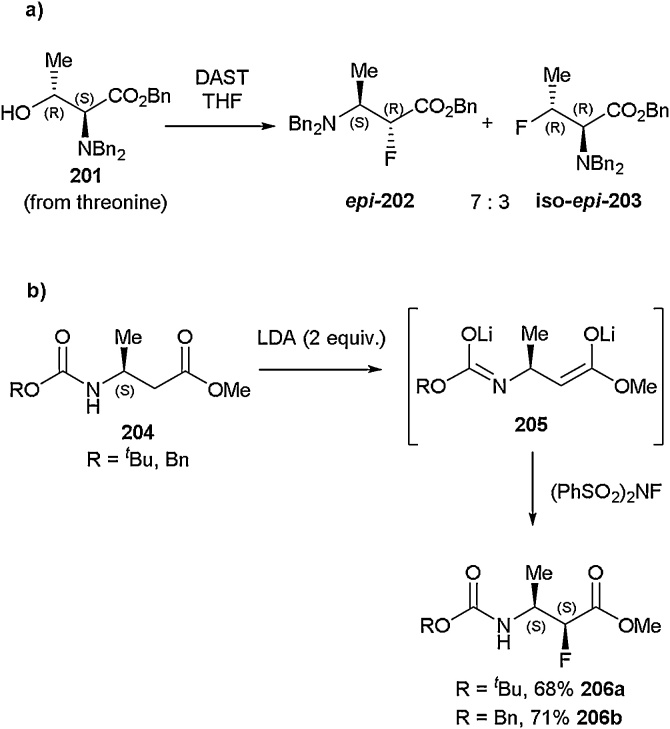
a) Synthesis of α-fluoro-β-amino acid derivatives, b) Synthesis of 3-amino-2-fluorobutanoates, Seebach and co-workers [144].

In addition, the group also reported the synthesis of butanoate derivative ***epi-*202** Bn through direct nucleophilic fluorination with DAST of threonine-derived benzyl ester **201** (Scheme 40a). A means for isolating Boc- and Cbz-(*S,S*)-3-amino-2-fluorobutanoates **206a-b** using two equivalents of LDA and an electrophilic source of fluorine in the form of (PhSO_2_)_2_NF was also described (Scheme 40b) [148,149].

**Scheme 41:**
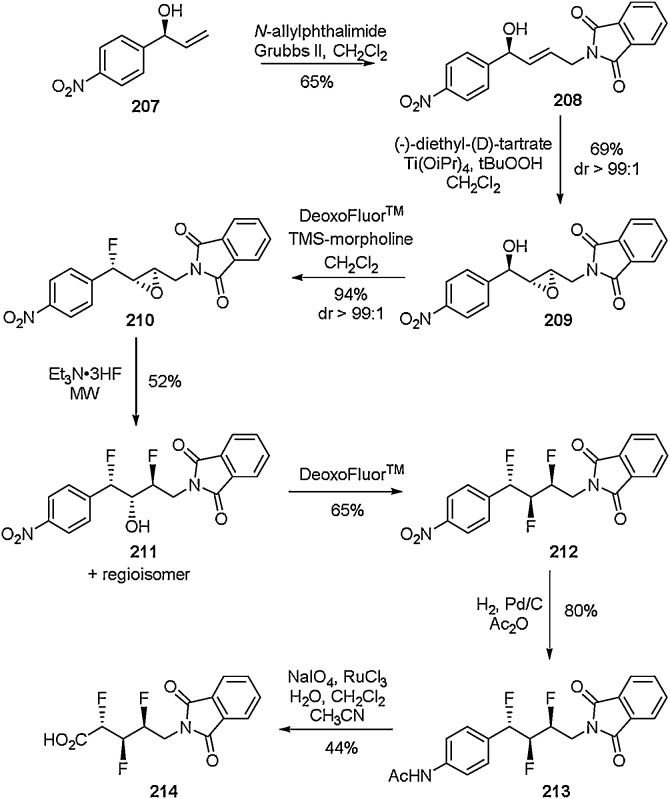
Synthesis of vicinal trifluoroamino acids, Hunter et al. [152].

From the compounds discussed in Schemes 35–38, it is clear to see that the ability to stereoselectively access acyclic fluorinated amino acids is extremely important. This selectivity becomes even more pressing when there is a desire to access vicinal multi-fluorinated systems. There have been several reports in which stereoselective vicinal multi-fluorinated amino acids have been accessed [150,151, [154,155]. Molecular systems which incorporate vicinal fluorine atoms have been shown to adopt unusual conformations. There have been several examples of amino acids with three vicinal fluorine atoms [150], and one such example is that of Hunter et al. who disclosed the synthesis of α,β,γ-trifluoro-*δ*-amino acids in 2013 using a sequential deoxyfluorination approach [152]. The *anti, syn* amino acid **214** was synthesised using a stereoselective epoxidation followed by deoxyfluorination sequence (Scheme 41). This synthetic route allowed the three contiguous fluorine atoms to be installed in a highly stereoselective manner. In the same report, the all-*syn* stereoisomer was also accessed using a variation on the synthetic strategy, using a Mitsunobu hydrolysis sequence to set the correct chirality of the backbone hydroxyl. Crystal structures of the anti, syn and all-syn regioisomers of compound **214** were obtained. These structures showed that the conformation each amino acid adopted was strongly influenced by the chirality of the installed fluorine atoms.

**Scheme 42:**
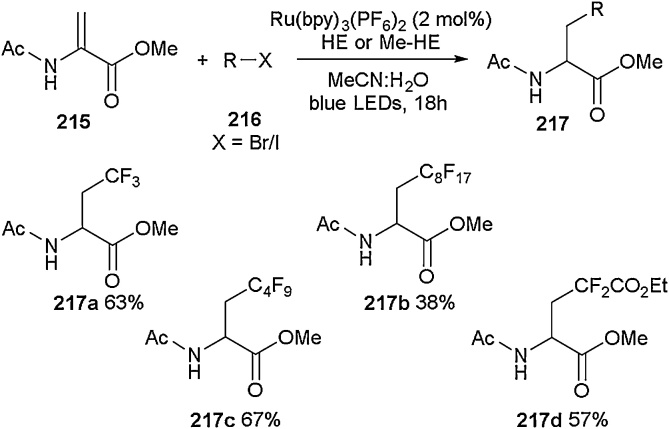
Photocatalysis to functionalise dehydroamino acids, Brandhofer et al. [160].

Olefin transformations have been exploited by several researchers to access a range of acyclic fluorinated amino acids [135,136,153], with hydration followed by fluorination being a well-established methodology applied in this area. In 2019, Brandhofer et al. developed a new approach with the use of ruthenium-based photocatalysis to functionalise dehydroamino acids (Scheme 42). This report complemented the previous iridium-based photocatalytic approaches using the Karady-Beckwith alkene [156,157]. It also supplemented previous work involving the employment of halopyridines [156], *N*-alkyl tertiary amines [157], potassium phenoxymethyl trifluoroborates [158] or imines [159]. Starting with the protected dehydroalanine **215**, coupling was conducted in the presence of a Ru(bpy)_3_(PF_6_)_2_ catalyst and Hantzsch ester (HE) or methyl Hantzsch ester (MeHE) with a range of alkyl radical precursors (alkyl iodides, bromides and *N*-(acyloxy)phthalimides). Using these conditions, a variety of fluorinated and perfluorinated alkyl-protected amino acids (**217a**-**d**) were obtained in moderate to good yield (38–67 %). This methodology was further extended to dehydro-2-methyl-β-alanine, dehydro-phenylalanine and dehydrobutyrine derivatives. In addition, it was also utilised in the late-stage functionalisation of an oligopeptide (thiostrepton) including the incorporation of fluorine atoms.

**Scheme 43:**
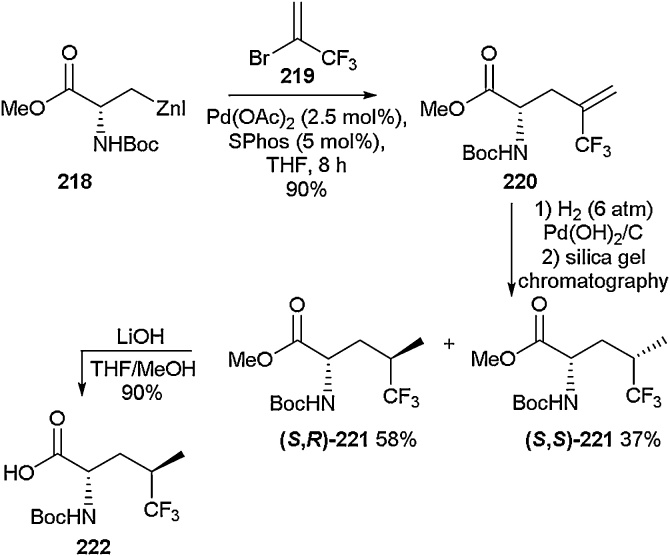
Negishi cross-coupling in the synthesis of trifluoromethyl alkene amino acids, Zhang and co-workers [161].

As well as being a target for addition reactions, alkenes can also be manipulated to provide other functionality. Zhang and co-workers utilised a Negishi cross-coupling approach using a 2-bromo-3,3,3-trifluoropropene fragment to access trifluoromethyl alkene amino acids that could be subjected to a range of further reactions (Scheme 43). For example, a Negishi cross-coupling reaction was carried out on the iodo-serine derivative **218** in order to access the trifluoromethyl alkene amino acid **220**. Using hydrogenation followed by chromatography to separate the diastereoisomers, **(*S*,*R*)-221** and **(*S,S*)-221** were isolated in 58 % and 37 % yields, respectively. The ester functionality was saponified under lithium hydroxide-mediated conditions to give the free carboxylic acid **222**, a substrate ready for further functionalisation.

### 3.3 SFx amino acids

**Scheme 44:**
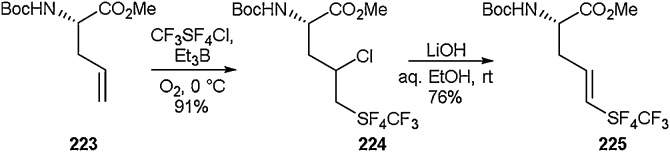
Access to an SF_4_CF_3_ alkene amino acid, Welch and co-workers [169].

Over the last decade there has been increasing interest in the synthetic community in accessing new S—F bond containing functionalities. With their unusual properties and the growing interest in SuFEx click chemistry, there has been a drive towards development of sulphur/fluorine-containing moieties [[162], [163], [164], [165], [166], [167]]. The addition of complex sulphur and fluorine containing functional groups across olefins has been studied by Welch and co-workers [168,169]. To a protected alkene-containing amino acid (**223**) was added CF_3_SF_4_Cl in the presence of base, allowing for an SF_4_CF_3_ moiety to be added across the alkene, giving compound **224** in 91 % (Scheme 44). This was then followed by elimination of the chlorine atom to give the final SF_4_CF_3_ alkene amino acid **225** in 76 % yield. Welch and co-workers had previously exploited this method to access the first pentafluorosulfanyl (SF_5_) containing amino acid in 2012 [168]. With these amino acid building blocks in hand, they were then further elaborated into short peptides and the influence that the fluorinated moiety had on the adopted secondary structure was probed.

**Scheme 45:**
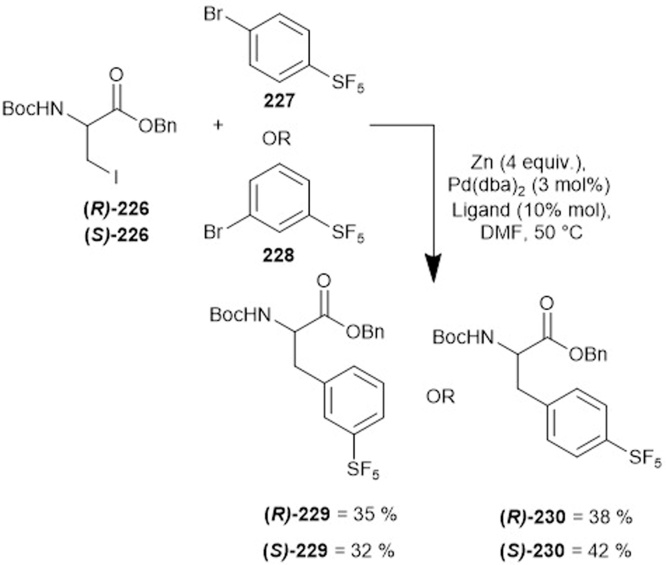
Aromatic SF_5_ amino acids, Cobb and co-workers [32].

Following on from Welch’s synthesis of alkenyl SF_5_-containing amino acids, Cobb and co-workers described the synthesis of pentafluorosulfanyl-containing aromatic amino acids in 2018 with a yield ranging from 32 to 42% (Scheme 45) [32]. The method employed a Negishi cross-coupling between the *R* or *S* isomer of halogenated amino acid precursor **226** and either pentaﬂuorosulfanyl-aryl **227** or **228** in the presence of zinc, an SPhos ligand (10 mol%) and a palladium catalyst. This led to the successful isolation of desired enantiopure compounds **229** or **230,** which could then undergo further selective deprotection at either the amine or carboxyl functionality for use in peptide coupling [31].

**Scheme 46:**
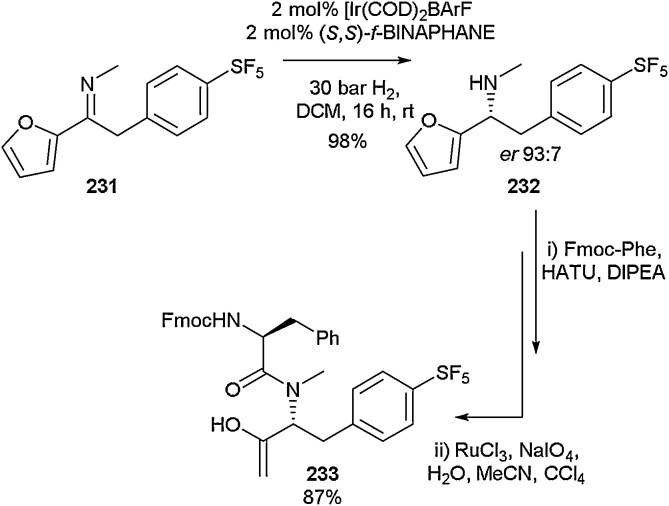
Synthesis of Fmoc SF_5_-containing dipeptide, Johansson and co-workers [170].

The synthesis of Fmoc-protected dipeptide **233** including an SF_5_ aromatic amino acid was reported by Johansson and co-workers in 2019 (Scheme 46). Initial enantioselective hydrogenation of pro-chiral *N*-alkyl α-aryl ketimine **231** gave chiral amine **232**. Subsequent coupling with Fmoc-protected phenylalanine under standard conditions followed by oxidative cleavage of the furyl group to the carboxylic acid in the presence of ruthenium trichloride and sodium periodate, afforded dipeptide **233**. The group also described the synthesis of other fluorinated phenylalanine analogues, including one possessing a CF_3_ group at the *para* position of the phenyl ring and another bearing a single fluorine atom at the same position.

## 4 Conclusions and outlook

In summary, the area of fluorinated amino acid synthesis has seen rapid growth over the past decade. It is also clear from the examples presented in this review that more complex fluorinated amino acids are increasingly being reported in the literature. A wide variety of aromatic and non-aromatic scaffolds have been accessed through a myriad of synthetic approaches. The development of palladium-catalysed cross coupling reactions and the emergence of strategies such as photoredox catalysis has seen an explosion in the types of moiety that can be incorporated into amino acids, and in turn, fluorine-containing functionality. As the fluorine chemistry community develop new functional groups, there will inevitably be much scope for the installation of these moieties into amino acids, and in turn peptides. For example, in the last decade we have seen the introduction of the SF_5_ and SF_4_CF_3_ functional groups to the peptide chemistry arena. The outlook for the field is strong, and as more synthetic techniques and moieties are introduced, the permutations of fluorine-containing amino acids to be targeted will increase, leading to vast potential for new scaffolds to be accessed.

## Declaration of Competing Interest

The authors report no declarations of interest.
